# Comparative Genome Analysis of Enterococcus cecorum Reveals Intercontinental Spread of a Lineage of Clinical Poultry Isolates

**DOI:** 10.1128/msphere.00495-22

**Published:** 2023-02-16

**Authors:** Jeanne Laurentie, Valentin Loux, Christelle Hennequet-Antier, Emilie Chambellon, Julien Deschamps, Angélina Trotereau, Sylviane Furlan, Claire Darrigo, Florent Kempf, Julie Lao, Marine Milhes, Céline Roques, Benoit Quinquis, Céline Vandecasteele, Roxane Boyer, Olivier Bouchez, Francis Repoila, Jean Le Guennec, Hélène Chiapello, Romain Briandet, Emmanuelle Helloin, Catherine Schouler, Isabelle Kempf, Pascale Serror

**Affiliations:** a Université Paris-Saclay, INRAE, AgroParisTech, Micalis Institute, Jouy-en-Josas, France; b ANSES Laboratoire de Ploufragan-Plouzané-Niort, Ploufragan, France; c Université Paris-Saclay, INRAE, BioinfOmics, MIGALE Bioinformatics Facility, Jouy-en-Josas, France; d Université Paris-Saclay, INRAE, MaIAGE, Jouy-en-Josas, France; e INRAE, Université de Tours, BOA, Nouzilly, France; f INRAE, Université de Tours, ISP, Nouzilly, France; g INRAE, Genotoul, GeT-PlaGe, Castanet-Tolosan, France; h Université Paris-Saclay, INRAE, MGP, Jouy-en-Josas, France; i Labofarm, Loudéac, France; University of Wisconsin—Madison

**Keywords:** *Enterococcus cecorum*, comparative genomics, avian pathogenesis, antimicrobial resistance, poultry, veterinary pathogens

## Abstract

Enterococcus cecorum is an emerging pathogen responsible for osteomyelitis, spondylitis, and femoral head necrosis causing animal suffering and mortality and requiring antimicrobial use in poultry. Paradoxically, E. cecorum is a common inhabitant of the intestinal microbiota of adult chickens. Despite evidence suggesting the existence of clones with pathogenic potential, the genetic and phenotypic relatedness of disease-associated isolates remains little investigated. Here, we sequenced and analyzed the genomes and characterized the phenotypes of more than 100 isolates, the majority of which were collected over the last 10 years from 16 French broiler farms. Comparative genomics, genome-wide association studies, and the measured susceptibility to serum, biofilm-forming capacity, and adhesion to chicken type II collagen were used to identify features associated with clinical isolates. We found that none of the tested phenotypes could discriminate the origin of the isolates or the phylogenetic group. Instead, we found that most clinical isolates are grouped phylogenetically, and our analyses selected six genes that discriminate 94% of isolates associated with disease from those that are not. Analysis of the resistome and the mobilome revealed that multidrug-resistant clones of *E. cecorum* cluster into a few clades and that integrative conjugative elements and genomic islands are the main carriers of antimicrobial resistance. This comprehensive genomic analysis shows that disease-associated clones of *E. cecorum* belong mainly to one phylogenetic clade.

**IMPORTANCE** Enterococcus cecorum is an important pathogen of poultry worldwide. It causes a number of locomotor disorders and septicemia, particularly in fast-growing broilers. Animal suffering, antimicrobial use, and associated economic losses require a better understanding of disease-associated *E. cecorum* isolates. To address this need, we performed whole-genome sequencing and analysis of a large collection of isolates responsible for outbreaks in France. By providing the first data set on the genetic diversity and resistome of *E. cecorum* strains circulating in France, we pinpoint an epidemic lineage that is probably also circulating elsewhere that should be targeted preferentially by preventive strategies in order to reduce the burden of *E. cecorum*-related diseases.

## INTRODUCTION

Enterococcus cecorum is a commensal bacterium of the gut microbiota of adult chickens (1–3). This bacterium has emerged over the last 20 years as a significant cause of locomotor disorders in poultry worldwide, particularly in fast-growing broilers (4). In France, reports on E. cecorum between 2006 and 2018 have shown an increase from 0.3% to >7% of total avian infections, the majority of which include locomotor disorders in broilers (5). *E. cecorum* is mostly responsible for osteomyelitis, spondylitis, vertebral osteoarthritis, and femoral head necrosis (1, 6, 7), causing substantial losses in broiler production due to culling, mortality, condemnations at the slaughterhouse, veterinary costs, and increased exposure to antibiotics (6, 8, 9). The development of *E. cecorum* infections is multifactorial and depends on host genetics, rapid growth, feed composition, husbandry procedures, and animal density in combination with the pathogenic potential of the bacterium (10–13). The association between the early intestinal carriage of *E. cecorum* and an increased risk of infections points to the gastrointestinal tract as a route of infection (10, 11, 14, 15). Recent studies indicate that lesion-associated *E. cecorum* isolates appear to be adapted to colonize the gut early in life, in contrast to nonclinical isolates (i.e., strains isolated from the gut of healthy birds), which do not colonize to a detectable level before week 3 (10, 14). It was also proposed that disinfection failure may contribute to *E. cecorum* persistence and outbreaks due to biofilm formation (7, 16–18). Although suggested by the prediction of host-binding proteins in the genome (19), adhesion to host tissue proteins has been overlooked, and the robustness of *E. cecorum* biofilms and associated properties remain to be investigated. It is also assumed that in Gram-positive bacteria, the thick layer of peptidoglycan that surrounds the cytoplasmic membrane confers resistance to the bactericidal activity of serum; for instance, human serum selectively kills commensal Enterococcus faecium strains, whereas disease-associated E. faecium strains are not susceptible (20). Assessing the pathogenic potential of *E. cecorum* isolates remains a challenge. Recently, an *in vivo* model has been used to compare the pathogenicity of two clinical isolates under field conditions, but it is applicable to only a limited number of strains (3). Less limiting, the chicken embryo lethality assay (CELA) has shown tendencies where pathogenic strains kill more efficiently than commensal isolates (14, 21).

Several molecular epidemiological studies based on pulsed-field gel electrophoresis (PFGE) patterns of commensal and clinical isolates from the United States, Canada, Belgium, the Netherlands, Germany, and Poland agree that commensal isolates have higher diversity than clinical isolates, suggesting the evolution of specific clones with greater pathogenic potential. However, clinical isolates exhibited multiple PFGE patterns, supporting the hypothesis of the polyclonal nature of the infectious isolates (22–26). Furthermore, repeated outbreaks with genotypically related isolates within farms and local areas substantiate horizontal transmission and a farm-related reservoir (7, 17, 24, 26, 27). To date, there are only 1 complete (type strain NCTC12421 [GenBank accession number NZ_LS483306.1]) and 84 partial nonredundant genomes of *E. cecorum* available. Only two comparative genomic studies of *E. cecorum* isolates from the United States have been performed (19, 28). A comparison of the partial genomes of three commensal and three clinical isolates from the southeastern United States isolated between 2010 and 2011 indicated that the pathogenic *E. cecorum* strains had smaller genomes with >120 genes absent or whose products had <40% identity in the commensal isolates (19). On the other hand, ~70 genes of the nonclinical isolates were absent or encoded products with <60% identity in the clinical isolates. In line with studies reporting a high rate of clinical isolates unable to metabolize mannitol (14, 24, 25), the orthologs of mannitol phosphate dehydrogenase, the mannitol operon activator, as well as mannitol-specific component IIA of the phosphotransferase system (PTS) were not found in clinical isolates (19). In another study, the partial genomes of nine clinical isolates isolated in Pennsylvania in 2008 and 2009 were compared with those of nine nonclinical isolates from the National Antimicrobial Resistance Monitoring System isolated between 2003 and 2010 (28). The trend of a slightly smaller genome size for clinical isolates was confirmed and was consistent with a larger accessory genome of nonclinical isolates. Noticeably, the nonclinical genomes had more antibiotic resistance genes. By combining available *E. cecorum* draft genome sequences (29, 30), the core genome was estimated to have 1,436 genes (28). Phylogenetic analysis of the core genome led the authors of this study to conclude that the isolates cluster independently of their clinical or nonclinical status, which raises the question of whether clinical isolates of *E. cecorum* belong to specific genetic groups. The objective of this study was to provide better insight into the genomic organization and phenotypic diversity of *E. cecorum* clinical isolates from broilers circulating between 2007 and 2017 in Brittany, the leading French commercial broiler-producing area. We performed whole-genome sequencing of more than 100 poultry and human clinical isolates in order to better define the extent of the genetic relatedness of clinical isolates and detect genes associated with virulence-related traits. We completed this genomic analysis by testing isolates for their adhesion to type II collagen, biofilm robustness, and growth in chicken serum. The overall genetic diversity of *E. cecorum* was investigated by pangenome analysis, with a particular focus on mobile genetic elements (MGEs), antimicrobial resistance genes (ARGs), and genome-wide association studies (GWASs) of the accessory genes and the phenotypic traits.

## RESULTS

### Clonality of *E. cecorum* clinical isolates.

To obtain insight into the gene repertoire of *E. cecorum*, we performed whole-genome sequencing of 118 isolates, including 100 clinical isolates collected from 16 broiler farms in western France between 2007 and 2017, 6 clinical isolates of human origin, and 8 clinical and 4 nonclinical isolates from other studies that had been previously sequenced (see Table S1 in the supplemental material). A total of 118 genomes sequenced by Illumina technology had sequencing coverage of >160× and an *N*_50_ of between 62 kbp and 276 kbp (Table S2A). Hybrid assemblies of Illumina and Nanopore data for 14 genomes allowed the reconstruction of 10 complete genomes (CIRMBP-1212, CIRMBP-1228, CIRMBP-1246, CIRMBP-1261, CIRMBP-1274, CIRMBP-1281, CIRMBP-1283, CIRMBP-1287, CIRMBP-1292, and CIRMBP-1302) and improvements of the genome assembly completeness of 4 others (Table S2A). The estimated average length of the genomes is ~2.4 Mb and varies between ~2.05 and ~2.8 Mbp. Each genome had an average of 2,345 predicted protein coding DNA sequences (CDSs). A total of 277,011 CDSs were annotated. Comparison of the chromosomal architecture of the 11 complete *E. cecorum* strains using the NCTC12421 strain as a reference revealed that strain CIRMBP-1261 has a large chromosomal inversion of ~1.8 Mbp between the second and sixth rRNA operons (Fig. S1). Strain CIRMBP-1287 also has a chromosomal inversion of 280 kbp from genes DQL78_RS05120 to DQL78_RS06585, involving an insertion sequence (IS) of the IS*3* family.

10.1128/msphere.00495-22.2 FIG S1 Inversion in two genomes compared to the reference genome. (A) Alignment of the CIRMBP-1261 and NCTC12421 genomes. (B) Alignment of CIRMBP-1287 and NCTC12421. Blue lines symbolize inversions in genomes. Download FIG S1, TIF file, 0.5 MB. Copyright © 2023 Laurentie et al. 2023 Laurentie et al. https://creativecommons.org/licenses/by/4.0/ This content is distributed under the terms of the Creative Commons Attribution 4.0 International license.

10.1128/msphere.00495-22.6 TABLE S1 Metadata of strains sequenced in this study. Download Table S1, XLSX file, 0.02 MB. Copyright © 2023 Laurentie et al. 2023 Laurentie et al. https://creativecommons.org/licenses/by/4.0/ This content is distributed under the terms of the Creative Commons Attribution 4.0 International license.

10.1128/msphere.00495-22.7 TABLE S2 (A) *E. cecorum* genomes sequenced in this study and included in the comparative analysis. (B) Nonredundant *E. cecorum* genomes available in the NCBI database included in the comparative analysis. Download Table S2, XLSX file, 0.03 MB. Copyright © 2023 Laurentie et al. 2023 Laurentie et al. https://creativecommons.org/licenses/by/4.0/ This content is distributed under the terms of the Creative Commons Attribution 4.0 International license.

**FIG 1:**
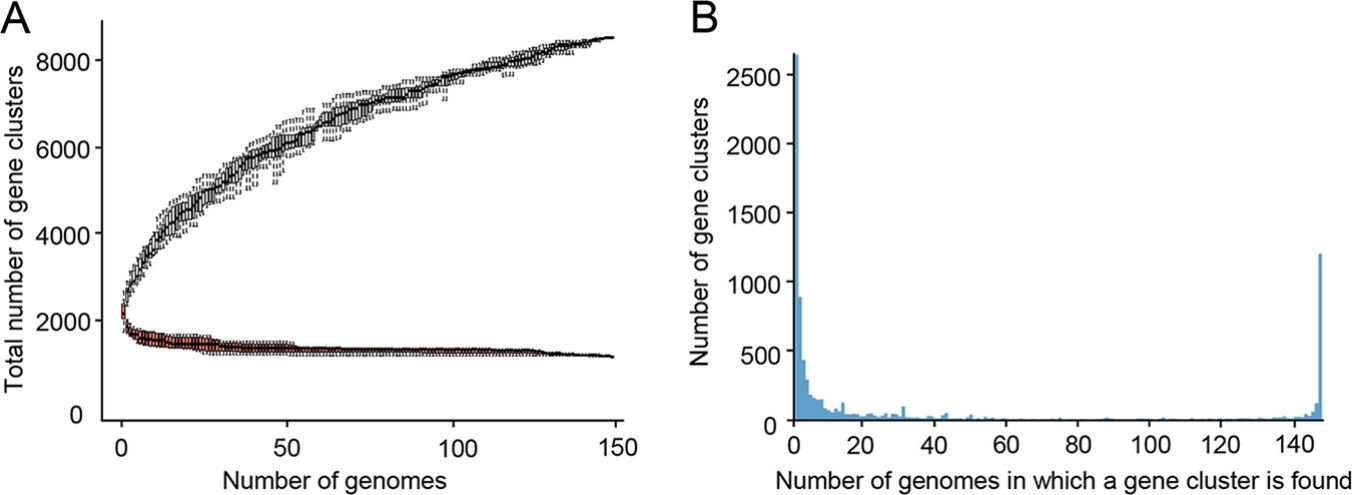
Pangenome analysis of 148 *E. cecorum* genomes. (A) Accumulation curves of gene clusters of the pan- and core genomes. The pangenome size, in black, corresponds to the total number of gene clusters against the number of genomes included. The core-genome size, in red, corresponds to the number of gene clusters in common against the number of genomes included; numbers were averaged on 1,000 randomized orders for genome addition. (B) Gene frequency spectrum. Only one representative per gene cluster is considered.

Thirty further nonredundant *E. cecorum* genomes available at the time of this work were included. These comprised genomes of 9 clinical and 21 nonclinical isolates from Belgium, Germany, and the United States (Table S2B). Comparative genomics analysis of the 351,733 CDSs from the 148 genomes identified 8,523 gene clusters in the pangenome, composed of a strict core genome (present in all *E. cecorum* genomes) of 1,207 CDSs, an accessory genome of 4,664 CDSs, and a unique genome of 2,652 (31.1%) CDSs (Fig. 1). The pangenome curve displayed an asymptotic trend after the 140-genome iteration, indicating the stabilization of the pangenome within this data set. Consistently, the core genome stabilized after the 125-genome iteration (Fig. 1A). These trends confirm that the genome data set used here provides a comprehensive overview of the gene repertoire of the *E. cecorum* species. The distribution of the gene clusters revealed that ~47% of the members of the pangenome (*n* = 3,979, including the unique genes) are present in one to three isolates (Fig. 1B), indicating that the genetic diversity is partly attributable to gene acquisition. This hypothesis is further supported by the high proportion (42%) of genes of unknown function.

**FIG 2:**
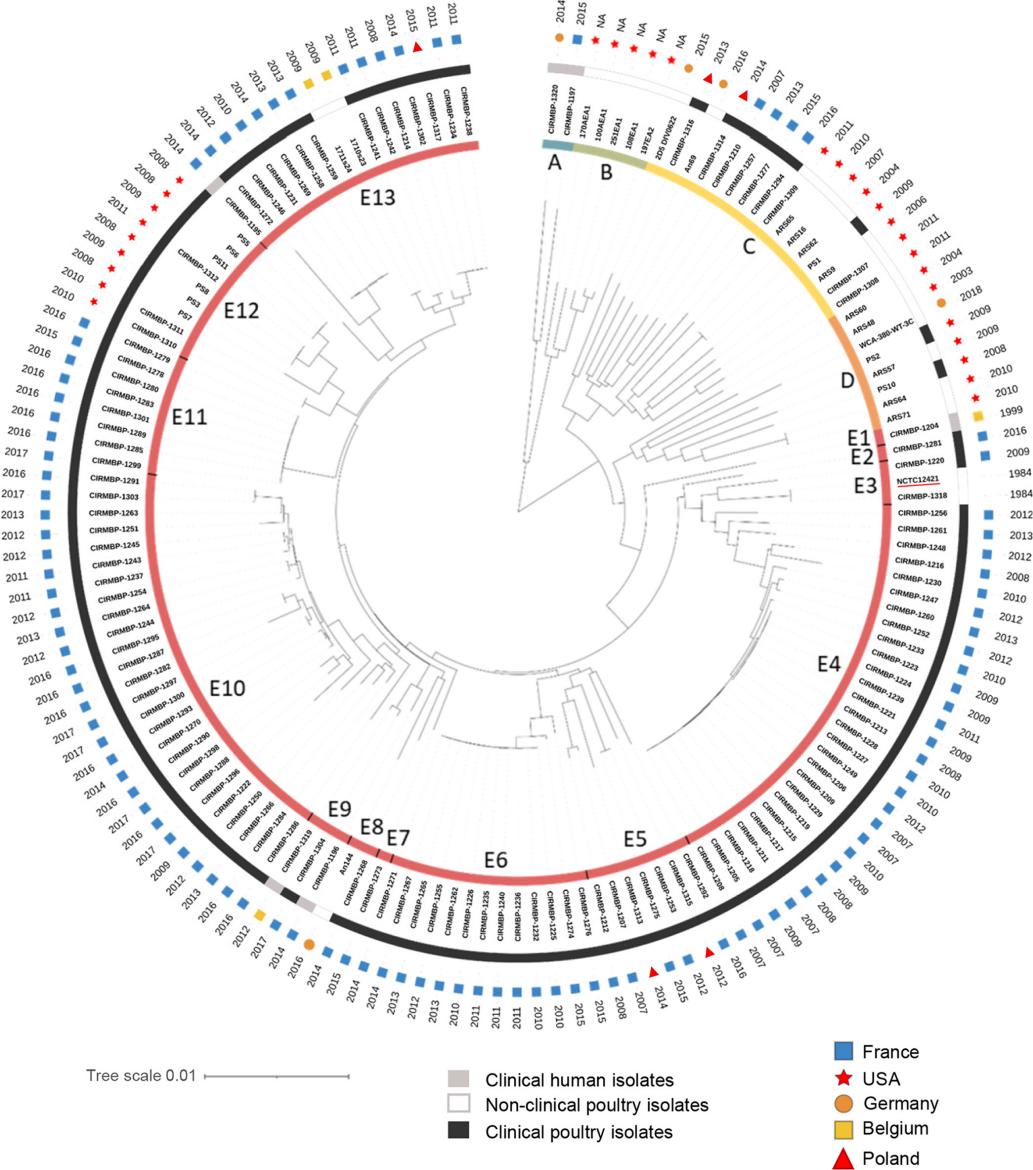
Phylogenetic tree and clinical status of 148 *E. cecorum* isolates. A neighbor-joining (BioNJ) tree was built on the pairwise distances between genomes. The innermost circle shows clades A to E with subclades E1 to E13 (colored strips). The first external circle shows the clinical status of the isolates. The second external circle shows the geographic origin. The third external circle shows the year of isolation. The name underlined in red corresponds to the reference genome of NCTC12421. Isolates CIRMBP-1307, CIRMBP-1308, CIRMBP-1309, CIRMBP-1310, CIRMBP-1311, CIRMBP-1312, CIRMBP-1313, CIRMBP-1314, CIRMBP-1315, CIRMBP-1316, and CIRMBP-1317 correspond to purified clones of strains CE1, CE2, CE3, SA1, SA2, SA3, CB-32, BB-66, CL-1, G-29, and D-104, respectively (see Table S1 in the supplemental material). NA, not applicable.

The *E. cecorum* neighbor-joining (BioNJ) phylogenetic tree was constructed using the concatenated sequence of the core genes (Fig. 2). The isolates clustered into five distinct phylogenetic clades (A to E), supported by a bootstrap value of 100% and the higher maximum pairwise genetic distance between clades (0.026 to 0.041) than within clades (<0.021) (Fig. S2). Clade E was further divided into 13 well-supported subclades (E1 to E13) with intrasubclade pairwise genetic distances of <0.012, indicating the greater clonality of these isolates. Clades A to D contain 2 to 16 genomes, only 35% of which were avian clinical isolates. While clades B and D contain mainly genomes of nonclinical isolates from the United States, clade A contains two European clinical human isolates, and clade C contains both nonclinical and clinical poultry isolates. In contrast, clade E comprises 117 genomes, 95% of which belong to avian clinical isolates from the United States, Belgium, Germany, Poland, and France, suggesting the widespread distribution of this clade. Of note, the type strain NCTC12421, isolated from the cecal contents of a dead chicken from a farm in Belgium (1, 31), is part of subclade E3, and subclade E12 contains only avian isolates from the United States. Although the number of isolates per subclade is limited, almost all isolates from subclades E6, E10, and E13 were isolated after 2009, while those from subclades E4 and E11 were isolated before 2014 and after 2015, respectively, indicating that the dominant subclasses have varied over the years. Due to sequence data availability, single nucleotide polymorphism (SNP) analysis was possible only for genomes sequenced in this study, thus excluding genomes from clades B and D and subclade E12. Of the 65,226 SNPs of the core genome, 2,443, 168, and 62 were specific to isolates of clades A (*n* = 2), C (*n* = 9), and E (*n* = 107), respectively. Of the 13 nonsynonymous clade E-specific SNPs, 8 are nonconservative, and 2 are predicted to be nonneutral in a phage shock protein (PspC [DQL78_RS04285 in NCTC12421]) and an ATP-binding cassette domain-containing protein (DQL78_RS04370 in NCTC12421).

10.1128/msphere.00495-22.3 FIG S2 Pairwise genetic distances between the *E. cecorum* genomes. The color scale goes from green for close genomes to red for distant genomes. Tree clades are framed with the respective colors used on the phylogenetic tree. Subclades are framed in black. Download FIG S2, TIF file, 1.0 MB. Copyright © 2023 Laurentie et al. 2023 Laurentie et al. https://creativecommons.org/licenses/by/4.0/ This content is distributed under the terms of the Creative Commons Attribution 4.0 International license.

Altogether, these data revealed that clinical isolates of *E. cecorum* from poultry from different countries are grouped phylogenetically and belong mainly to clade E, confirming their clonality and suggesting either the dissemination of well-adapted clones or convergent selection/adaptation to poultry genetics and breeding methods.

### Six genes significantly associated with origin differentiate avian clinical from nonclinical isolates in the 148 genomes.

To further explore the genetic basis for the epidemic success of the *E. cecorum* clade E isolates, we searched for genes significantly associated with clade membership. The majority of unique genes (71.9%) are carried by isolates from clades A, B, C, and D, indicating higher genetic diversity in these clades. However, this trend may result from the enrichment of clade E isolates in the collection. Consistently, we identified 97 accessory genes significantly associated with clade E isolates (Table S3A). The most abundant group of the 83 clade E-enriched genes after the unclassified hypothetical protein-encoding genes (*n* = 17) are predicted carbohydrate metabolism and transport genes (*n* = 13) and cell wall/membrane/envelope biogenesis genes (*n* = 12). A high proportion of the clade E-enriched genes are clustered loci, including the capsular polysaccharide (CPS) biosynthesis locus (CIRMBP1228_00568 to CIRMBP1228_00581 in strain CIRMBP-1228), a large gene cluster comprising biotin biosynthesis genes (CIRMBP1228_01807 to CIRMBP1228_01837), and one putative operon of carbohydrate metabolism (CIRMBP1228_02727 to CIRMBP1228_02733). Alignment of the gene products of the representative capsule biosynthesis loci from the complete genomes identified two closely related loci represented by the genomes of CIRMBP-1228 and CIRMBP-1212 (Fig. S3), which account for 66.7 and 14.5% of the clade E isolates, respectively. The remaining 14 genes significantly associated with the origin of the isolates (clinical and nonclinical poultry isolates or clinical human isolates) are predominantly absent in clade E isolates. They include genes encoding a pseudouridine synthase (CIRMBP1294_00547), a predicted nucleotide sugar dehydrogenase (CIRMBP1294_00386), a diacylglycerol kinase family lipid kinase (CIRMBP1294_00623), and two stress-related proteins (CIRMBP1294_00560 and CIRMBP1294_00758). In line with the large proportion of clade E clinical isolates in the collection, 30 clade E-enriched genes are also enriched in clinical poultry isolates compared to nonclinical poultry and clinical human isolates (Table S3B). These include genes of the CPS biosynthesis locus (CIRMBP1228_00573, CIRMBP1228_00574, and CIRMBP1228_00575), 27 genes of the biotin gene cluster, and an H protein gene of the glycine cleavage system generally involved in protein lipoylation. A total of 65 genes are significantly associated with the origin of the isolates. In addition to those enriched in clade E isolates, other genes enriched in avian clinical isolates encode a phosphoenolpyruvate:carbohydrate phosphotransferase system (PTS), a transketolase (CIRMBP1228_00604 to CIRMBP1228_00607), as well as proteins of unknown function. Although no specific gene signature for avian clinical isolates was found, we identified 6 accessory genes (CIRMBP1228_00573, CIRMBP1228_00586, CIRMBP1228_00757, CIRMBP1228_01816, CIRMBP1228_02735, and CIRMBP1283_01819) that allowed the identification of 94% of avian clinical isolates (Table S3C). With the exception of 7 avian clinical isolates from clade C or D, all other genomes of clinical isolates have an average of 4 of the 6 genes mentioned above (range, 2 to 6 genes), while nonclinical isolates have 1 gene at most. We also found 12 genes that may be enriched in clinical human isolates. These include 8 genes predicted to be involved in the import and utilization of ascorbate under anaerobic conditions (32, 33).

10.1128/msphere.00495-22.4 FIG S3 Alignment of the predicted capsule loci in the complete genomes. Arrows are annotated CDSs on both elements. The blue-scale shading represents regions of nucleotide sequence identity (100% to 94%) determined by BLASTN analysis. The number of isolates that share each type of locus, among the 117 other genomes in the study, is shown on the right. Download FIG S3, TIF file, 1.4 MB. Copyright © 2023 Laurentie et al. 2023 Laurentie et al. https://creativecommons.org/licenses/by/4.0/ This content is distributed under the terms of the Creative Commons Attribution 4.0 International license.

10.1128/msphere.00495-22.8 TABLE S3 (A) Genes differentially distributed between clade E isolates and isolates of other clades. (B) Genes differentially distributed between clinical and nonclinical poultry isolates or clinical human isolates. (C) Proposed set of discriminatory accessory genes. (D) Genes present in highly and intermediately virulent *E. cecorum* isolates in embryonated eggs and absent in nonvirulent isolates. Download Table S3, XLSX file, 0.2 MB. Copyright © 2023 Laurentie et al. 2023 Laurentie et al. https://creativecommons.org/licenses/by/4.0/ This content is distributed under the terms of the Creative Commons Attribution 4.0 International license.

Despite the difficulty in identifying genes specific to the origin of the isolates, this work highlights six genes encoding a glycosyltransferase (CIRMBP1228_00573), two PTS EIIC components (CIRMBP1320_01424 and CIRMBP1228_02735), and three hypothetical proteins (CIRMBP1228_00586, CIRMBP1228_01816, and CIRMBP1228_00757) that can discriminate most of the isolates associated with poultry disease from those that are not.

### Multiresistant clones of *E. cecorum* cluster into a few clades.

**FIG 3:**
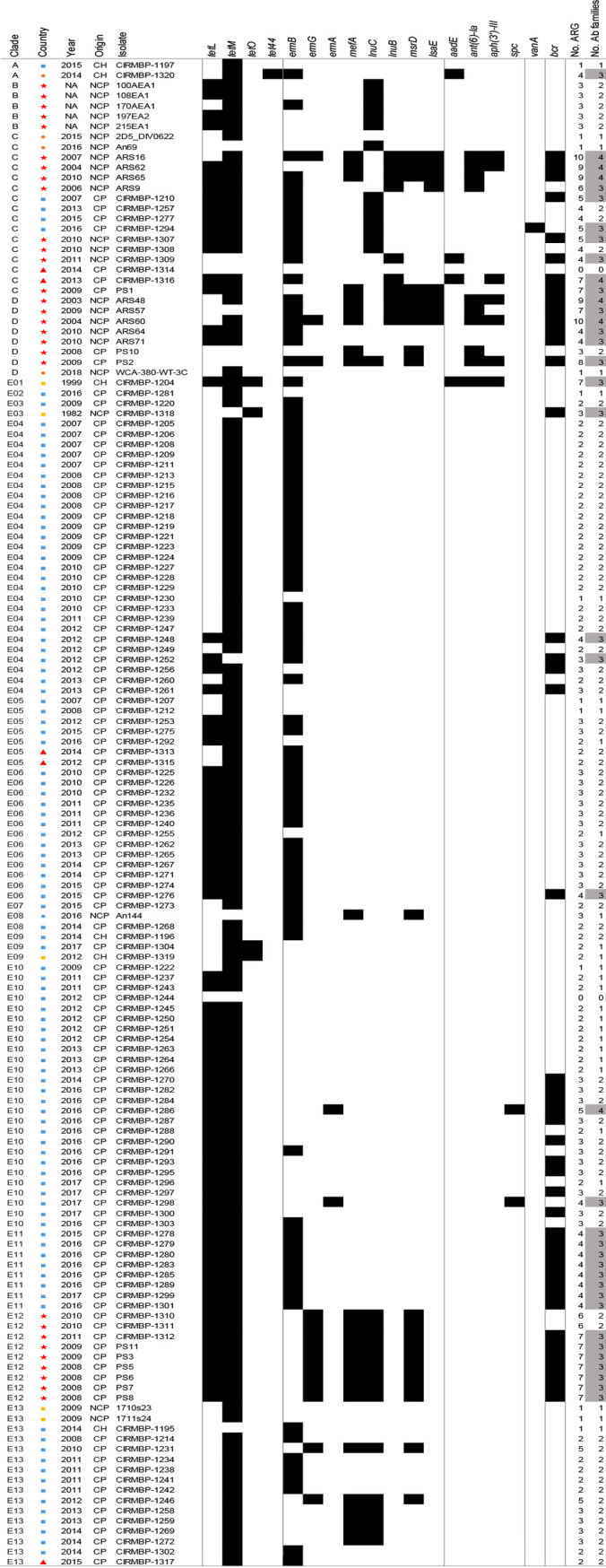
Distribution of antimicrobial resistance genes in sequenced genomes. The clade, geographic origin (blue squares, France; orange circles, Germany; red stars, United States; red triangles, Poland; yellow squares, Belgium), isolation year, and clinical origin of the sample (CH, clinical human; NCP, nonclinical poultry; CP, clinical poultry) are specified. The following antibiotic (Ab) families are represented by alternating gray blocks, from left to right: tetracycline, MLS (macrolide, lincosamide, and streptogramin), aminoglycoside, glycopeptide, and bacitracin. Black strips represent the presence and white ones represent the absence of ARGs. Potential multiresistant isolates (>2 ARGs for different families) are highlighted in gray.

Next, we searched the 148 sequenced genomes for the presence of acquired ARGs using ResFinder, BLAST, and the PLSDB database. A total of 18 ARGs were identified (Fig. 3). Tetracycline and macrolide-lincosamide-streptogramin B (MLS) resistance genes were detected in 95% and 75% of the isolates, respectively. In total, 70% of the genomes had at least one gene for resistance to these two families of antimicrobials, with *tet*(M) and *erm*(B) being the most prevalent. Genes for resistance to other antimicrobial classes such as aminoglycosides (10%), bacitracin (30%), and vancomycin (0.6%) were also detected. Only two isolates (CIRMBP-1244 and CIRMBP-1314) did not contain any ARG searched. Overall, 39 genomes carried genes conferring resistance to three antimicrobial families, and 7 genomes had at least four genes for resistance to different antimicrobial families (Fig. 3). The most common combinations of ARGs in multidrug-resistant isolates cover the tetracycline and MLS families in combination with aminoglycosides in clades C and D or with bacitracin in clades C and D and subclades E10, E11, and E12. Aminoglycoside resistance genes are carried by nonclinical genomes with enrichment in U.S. strains. The near-systematic presence of ARGs in the genomes of *E. cecorum* isolates suggests that the species may have undergone strong selection for antibiotics, tetracyclines and MLS in particular, but that resistance to these two antibiotic families did not provide a selective advantage to clinical isolates.

Several *E. cecorum* genome regions carrying ARGs conserved in Gram-positive bacteria were identified in the PLSDB database, but none were carried by a previously identified plasmid. This analysis highlighted different ARG clusters. They comprised the *erm*(B), *vat*(D), and *msr*(D) genes; the *tet*(L), *tet*(M), and *bcr* genes; or the *tet*(L), *tet*(M), *ant(6)*, and *bcr* genes. Further characterization of the vancomycin resistance operon carried by strain CIRMBP-1294 revealed that the *vanA* operon was integrated into the chromosome 12 kbp away from the genes that confer resistance to narasin and erythromycin in between (Fig. S4). This chromosomal region was closely related to that of the E. faecium plasmid pVEF3 (34) identified in broiler isolates from Sweden (35) and was flanked by two transposase genes of the IS*3* family. However, no conjugative or mobilization element was detected close to this ARG cluster. Although about 20 plasmid-related gene clusters were identified in the pangenome, a single putative 4.4-kbp plasmid was detected in the complete genome of strain CIRMBP-1292 and 6 other isolates (CIRMBP-1233, CIRMBP-1259, CIRMBP-1286, CIRMBP-1289, and CIRMBP-1304), but no ARG was detected on this small plasmid. Together, these results suggest that plasmids are rare in this bacterial collection and that ARGs are located on mobile genomic islands (GIs).

10.1128/msphere.00495-22.5 FIG S4 Alignment of vancomycin and narasin resistance operons between CIRMBP-1294 and E. faecium plasmid pVEF3. Blue arrows are annotated CDSs on both elements. Blue highlighting represents ≥99% identity by BLASTN analysis. Frames indicate vancomycin resistance and narasin resistance operons. Download FIG S4, TIF file, 0.7 MB. Copyright © 2023 Laurentie et al. 2023 Laurentie et al. https://creativecommons.org/licenses/by/4.0/ This content is distributed under the terms of the Creative Commons Attribution 4.0 International license.

### The *E. cecorum* mobilome is a major contributor to inter- and intraclade diversity.

**FIG 4:**
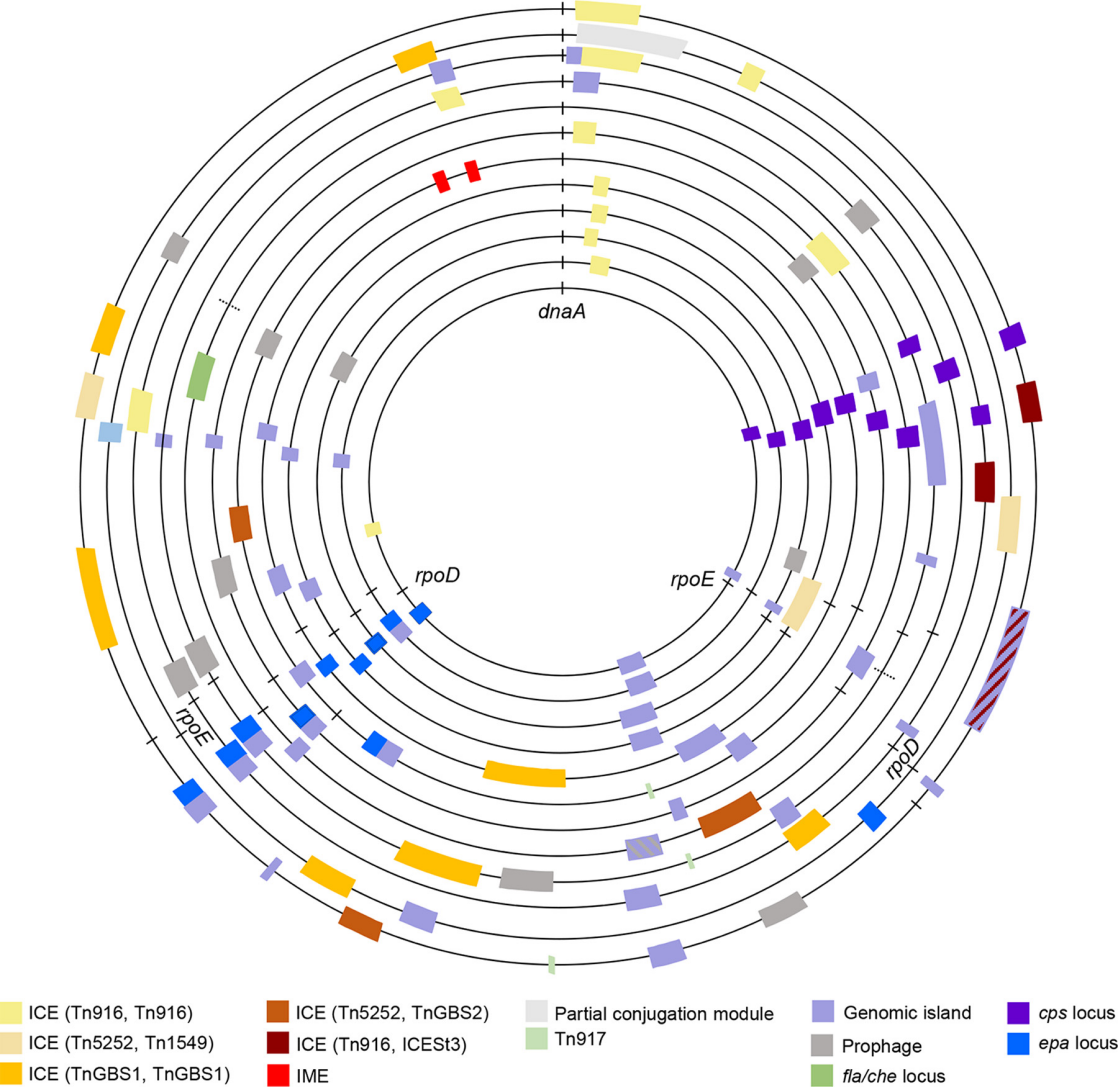
Mobile genetic elements in complete *E. cecorum* genomes. From the inner to outer circles are CIRMBP-1212, CIRMBP-1283, CIRMBP-1292, CIRMBP-1246, CIRMBP-1302, NCTC12421, CIRMBP-1287, CIRMBP-1320, CIRMBP-1274, CIRMBP-1281, CIRMBP-1261, and CIRMBP-1228. The dotted lines on the genome of CIRMBP-1320 correspond to predicted junctions. Each colored rectangle indicates the integration of an element according to the color key. Hatching indicates a complex genomic island. The *dnaA*, *rpoE*, and *rpoD* genes and the *epa* and *cps* loci are indicated.

In Gram-positive bacteria, ARGs and related transposons are frequently integrated into complex MGEs forming integrative conjugative elements (ICEs) or integrative mobilizable elements (IMEs) that may be difficult to identify in draft genomes (36). To evaluate the contribution of these elements to the spread of ARGs and *E. cecorum* genome diversity, we predicted ICEs and IMEs in the 11 complete genomes and the 2 large contigs of the genome of CIRMBP-1320 using ICEscreen (37). ICEscreen ICE and IME detection relies on the presence of signature protein CDSs (integrase, coupling protein, relaxase, and VirB4) grouped on the genomes and previously shown to be a valuable clue to the presence of an integrative element (38, 39). ICEs were defined by the superfamily and family of their signature proteins (38). Each *E. cecorum* genome contained at least one ICE (Fig. 4 and Table S4A). In total, 33 mobile genetic elements, including 5 types of ICEs (belonging to the Tn*916*, Tn*5252*, and Tn*GBS1* superfamilies), 2 IMEs, 3 Tn*917* elements, and 1 partial conjugative element, were identified. Their sizes ranged from ~5 kbp for Tn*917* to ~103 kbp for the Tn*GBS1* ICE of the genome of CIRMBP-1302. The Tn*916* ICE of the genomes of CIRMBP-1228 and CIRMBP-1281, integrated upstream of the 30S ribosomal protein S6-encoding gene, included another ICE of an undescribed family encoding a DDE transposase, a MobC-like relaxase, a VirD4-like coupling protein, and a type IV secretion system (T4SS) protein, VirB4. We also investigated the presence of other GIs in the complete genomes (Fig. 4 and Table S4B). A total of 42 GIs were identified, with sizes ranging from ~8 kbp to ~122 kbp for the complex GI that comprises an ICE (Tn*916*, ICE*Bs1*, and ICE*St3*) in the genome of CIRMBP-1228. Each genome harbored from 3 ICE-related elements or genomic elements (strain CIRMBP-1212 of subclade E5) up to 12 (strain CIRMBP-1228 of subclade E4). They were mainly integrated into intergenic regions or the 3′ ends of genes, with no effect on the encoded sequences. As expected, all Tn*917* elements and ICEs of the Tn*916* family carried *erm*(B) and *tet*(M), respectively. Other ARGs, such as *tet*(L), *tet*(O), *ant(6)*, and the *bcr* operon, were carried on Tn*916*-related ICEs or on GIs. Besides genes involved in GI transfer, a few had predicted functions related to biotin biosynthesis, restriction-modification enzymes, cadmium/arsenate resistance, toxin-antitoxin systems, redox enzymes, type 2 secretion systems, and flagella and chemotaxis. Analysis of the distributions of the ICE-related elements and the GIs in the other 136 *E. cecorum* genomes (Table S4B) revealed three highly dispersed elements: the Tn*916*-related ICE of strain CIRMBP-1292 in 113 genomes, the GI inserted near the *rpsB* gene and carrying the biotin biosynthesis genes found in 90 genomes, and the transposon Tn*917* in 65 genomes (Table S4B). The GI inserted between *dnaX* and *sufB* of strains CIRMBP-1287 and CIRMBP-1274 was highly prevalent in subclades E6 and E10, respectively. The same is true for the cognate element of CIRMBP-1283 and CIRMBP-1302 that is detected in all isolates of subclade E11, to which CIRMBP-1283 belongs. Other elements are enriched in specific subclades, such as the GI near the 23S rRNA methyltransferase gene *rlmD* and ICEs of the Tn*GBS1* and Tn*916* families of CIRMBP-1274 enriched in subclade E6, the ICE of the Tn*916* family of CIRMBP-1212 enriched in subclade E13, and the GI inserted at the 3′ end of CIRMBP1228_01030 and the ICE of the Tn*GBS2* family of CIRMBP-1228 close to gene CIRMBP1228_01622 enriched in subclade E4. According to their gene contents, 15 elements appear to be more strain specific in strains CIRMBP-1320 (*n* = 4), CIRMBP-1281 (*n* = 4), CIRMBP-1292 (*n* = 3), and NCTC12421 (*n* = 4). Although the genomes used to identify ICEs and GIs are not representative of all clades, homologs were found in almost all clades except for clade B, which gathers only five nonclinical poultry isolates from the United States. However, these genomic elements were less conserved in isolates from the United States, suggesting that they were acquired independently. We conclude that the majority of the identified ICEs and GIs are enriched in some clades, while only a few are shared across clades.

10.1128/msphere.00495-22.9 TABLE S4 (A) Transposons, ICEs, IMEs, and genomic islands detected in *E. cecorum* complete or high-quality genomes. (B) Distribution of ICEs and genomic islands of 12 strains in all available *E. cecorum* genomes. Download Table S4, XLSX file, 0.04 MB. Copyright © 2023 Laurentie et al. 2023 Laurentie et al. https://creativecommons.org/licenses/by/4.0/ This content is distributed under the terms of the Creative Commons Attribution 4.0 International license.

As prophages are easier to detect than GIs and mobile genetic elements, prophages were searched for in all *E. cecorum* genomes sequenced, including the genome of NCTC12421. Totals of 103 complete prophages and 30 incomplete prophages were identified in 78 (66.1%) *E. cecorum* genomes (Table S5). The prophage size ranged between 31 and 59 kbp. The prophages were related to 19 different types that varied across the phylogenetic groups. Subclade E4 was enriched with prophages related to *Faecalibacterium* phage oengus (40), which was the most commonly found prophage (*n* = 31), followed by prophages related to the *Siphoviridae* temperate phage EFC-1 of Enterococcus faecalis (41) (*n* = 16). Prophages related to the *Myoviridae* temperate bacteriophage EJ-1 of Streptococcus pneumoniae (42) (*n* = 13) were found mostly in the genomes of subclade E10, while those related to the *Siphoviridae* temperate phage 50101 of Lactococcus (*n* = 12) were most prevalent in subclade E6. Conversely, clade C had the most diverse prophages, and subclades E10 and E12 contained the smallest number of prophages. Prophages related to *Siphoviridae* temperate phage 5093 of Streptococcus thermophilus (43) (*n* = 9) were identified in several phylogroups. A total of 13 different integration sites for temperate bacteriophages were identified, mainly in intergenic regions and at the 3′ ends of genes, without changing the reading frame (Table S5). However, the prophage related to PHAGE_Strept_EJ_1_NC_005294 integrated into the PreQ1 riboswitch is likely to change the expression of the downstream nucleoside hydrolase gene. Like ICEs and GIs, prophages are less prevalent in the ~700 kbp surrounding the origin of replication of the chromosome (Fig. 4), indicating that MGEs are not randomly distributed in the *E. cecorum* genome.

10.1128/msphere.00495-22.10 TABLE S5 (A) Prophages predicted in *E. cecorum* genomes sequenced in this study. (B) *E. cecorum* genomes with no predicted prophages. Download Table S5, XLSX file, 0.04 MB. Copyright © 2023 Laurentie et al. 2023 Laurentie et al. https://creativecommons.org/licenses/by/4.0/ This content is distributed under the terms of the Creative Commons Attribution 4.0 International license.

### Biofilm robustness, adhesion to type II collagen, and growth in chicken serum are not associated with gene content.

**FIG 5:**
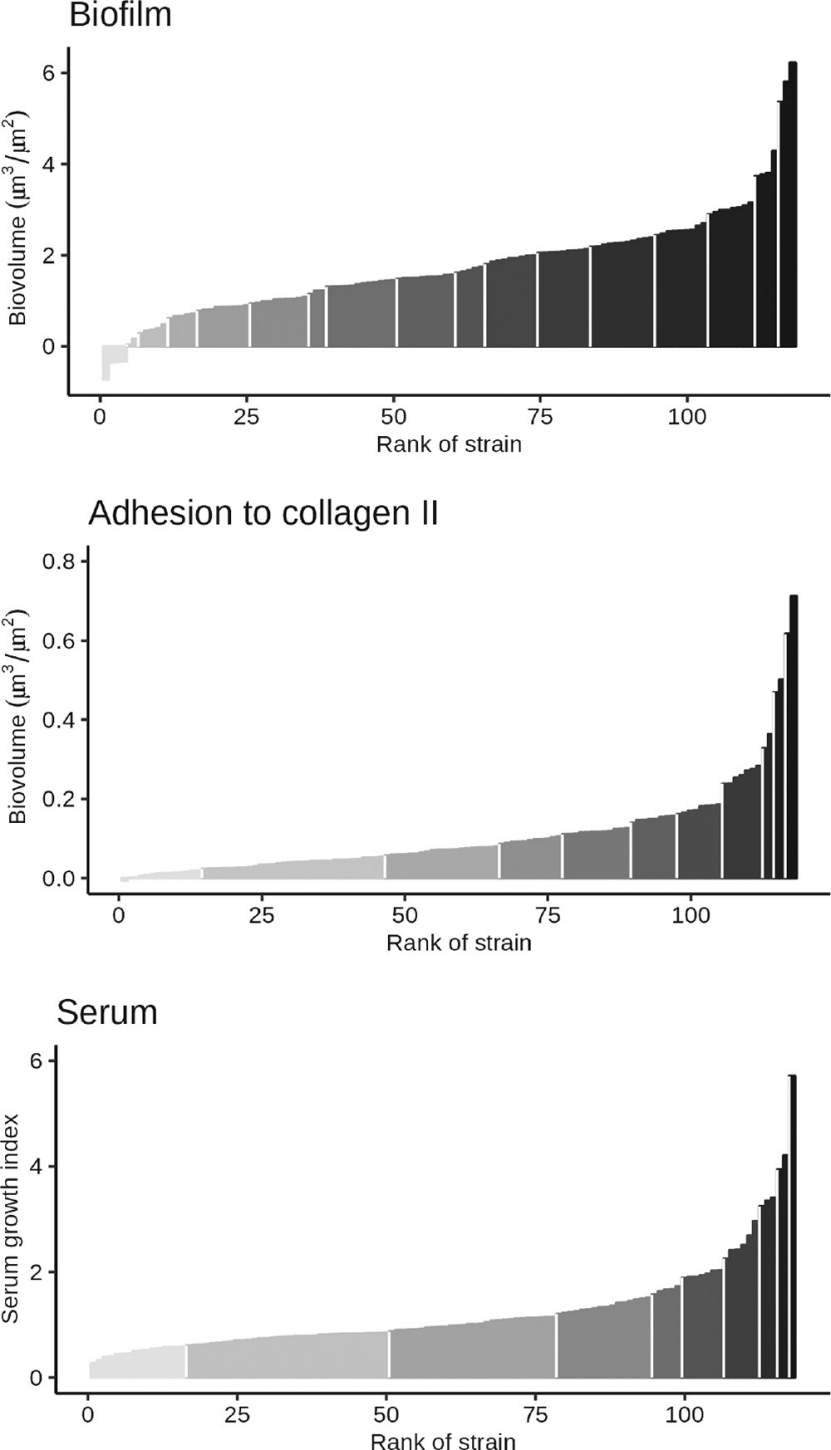
Distribution of phenotypic expression for biofilm robustness, adhesion to type II collagen, and growth in chicken serum for 118 *E. cecorum* isolates. Values for biofilm and adhesion to collagen represent estimated marginal mean biovolumes. Values for growth in serum represent estimated marginal means of serum growth indices calculated at 6 h (see the supplemental material). Each bar corresponds to an isolate. Strains with similar phenotypic expression were grouped into clusters on a gray color scale, separated by white lines.

To obtain phenotypic insight into the 118 isolates, we independently examined phenotypes relevant to *E. cecorum* pathogenesis: biofilm robustness, adhesion to type II collagen, and growth in chicken serum (see Text S1 in the supplemental material). Hierarchical clustering of these phenotypes (Fig. 5) revealed 17, 11, and 9 groups of strains for biofilm robustness, type II collagen adhesion, and growth in chicken serum, respectively. The lack of concordance between these phenotypic groups and clades strongly suggests that none of the phenotypes discriminate between clades. In an attempt to rank isolates, the most robust biofilm-forming isolates were CIRMBP-1302 (subclade E13), CIRMBP-1277 (clade C), and CIRMBP-1228 (subclade E4), and the most fragile biofilm-forming isolates were CIRMBP-1204 (subclade E1), CIRMBP-1205 and CIRMBP-1211 (subclade E4), and CIRMBP-1225 (subclade E6). The most collagen-adherent isolates were CIRMBP-1274 (subclade E6) and CIRMBP-1277 (clade C). All of the *E. cecorum* isolates had a serum growth index below the index of the Escherichia coli control strain sensitive to the bactericidal effect (190.34 ± 123.88) of serum and in the range of that of the nonsensitive E. coli control strain (3.172 ± 4.2). The *E. cecorum* isolates with the lowest indices were strains CIRMBP-1318 (0.229 ± 0.21) and CIRMBP-1206 (0.467 ± 0.001), belonging to subclades E3 and E4, respectively. The *E. cecorum* strains with the highest indices were strains CIRMBP-1312 (3.85 ± 0.44) of subclade E12, CIRMBP-1197 (4.21 ± 2.2) of clade A, and CIRMBP-1298 (5.71 ± 2.9) of subclade E10. Taken together, these results indicate that the growth of *E. cecorum* is not affected by the presence of chicken serum. Besides strain CIRMBP-1277, which formed strong biofilms and had a high capacity to adhere to collagen, no relationship between the expression of the three phenotypes and the strains was observed. Moreover, no accessory genes were associated with the phenotypic groups or with binary transformed values, suggesting functional redundancy between genes and/or differential gene expression between isolates.

10.1128/msphere.00495-22.1 TEXT S1 Supplemental methods. Download Text S1, PDF file, 0.2 MB. Copyright © 2023 Laurentie et al. 2023 Laurentie et al. https://creativecommons.org/licenses/by/4.0/ This content is distributed under the terms of the Creative Commons Attribution 4.0 International license.

### Clinical isolates of *E. cecorum* show a broad spectrum of virulence in chicken embryos.

**FIG 6:**
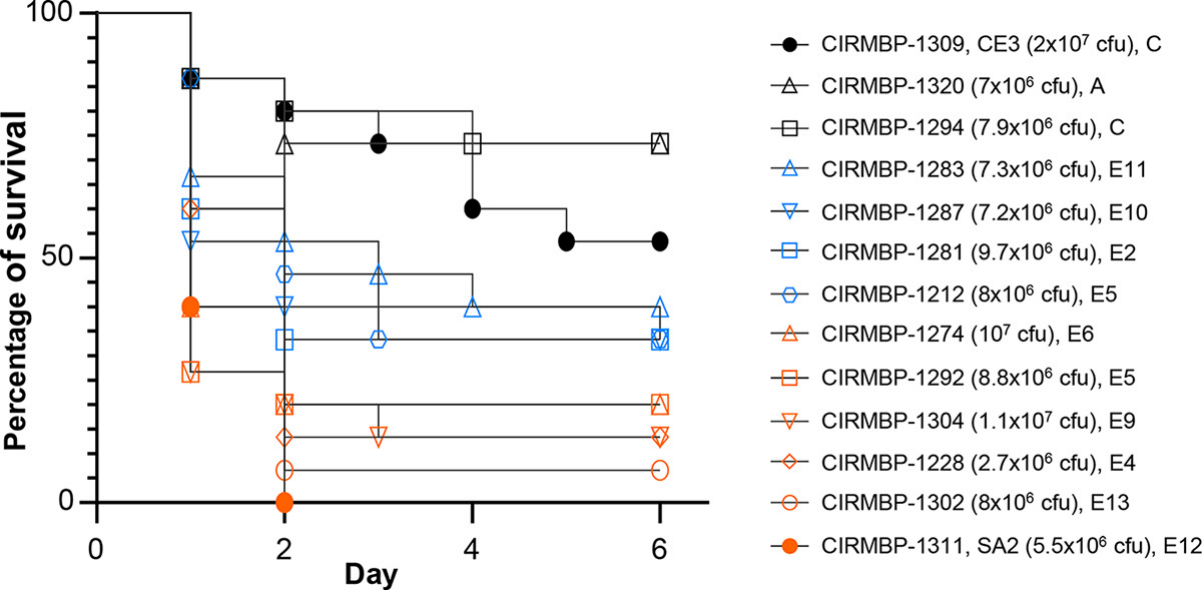
Comparison of the virulence of selected *E. cecorum* isolates in a chicken embryo model of infection. Shown is a Kaplan-Meier survival plot of chicken embryos (*n* = 15) infected with 13 different isolates of *E. cecorum*. The strain name, inoculum size, and phylogenetic group are indicated. The log rank (Mantel-Cox) test indicated significant differences between the positive-control strain CIRMBP-1311 (SA2) and strains CIRMBP-1309 (CE3) (*P* < 0.0001), CIRMBP-1212 (*P* = 0.0007), CIRMBP-1281 (*P* = 0.0364), CIRMBP-1283 (*P* = 0.0035), CIRMBP-1287 (*P* = 0.0339), CIRMBP-1294 (*P* < 0.0001), and CIRMBP-1320 (*P* < 0.0001). The virulence of strains CIRMBP-1292, CIRMBP-1304, CIRMBP-1228, CIRMBP-1274, and CIRMBP-1302 was not significantly different from that of CIRMBP-1311. Data from one representative experiment of two are shown.

To evaluate the pathogenic potential of *E. cecorum* strains isolated from diseased broilers in French farms, we used the chicken embryo lethality assay (CELA) reported previously by Borst et al. using strain CIRMBP-1309 (original name, CE3) and strain CIRMBP-1311 (original name, SA2) as biological controls for a nonpathogenic and pathogenic poultry isolate, respectively (21). We tested the 11 *E. cecorum* strains with the most complete genomes (see Text S1 in the supplemental material). No embryo died in the control group. The two *E. cecorum* control strains behaved as expected: 53% of the embryos were still alive 6 days after inoculation with the commensal strain CIRMBP-1309, whereas 100% of the embryos died after 2 days when inoculated with strain CIRMBP-1311 isolated from spondylitis lesions (Fig. 6). Strain CIRMBP-1294, isolated from infected vertebrae, and strain CIRMBP-1320, isolated from human infection, induced <27% embryonic lethality, indicative of low virulence in the CELA. In contrast, strains CIRMBP-1228, CIRMBP-1274, CIRMBP-1292, CIRMBP-1302, and CIRMBP-1304, isolated mainly from infected vertebrae, showed the same virulence as that of strain CIRMBP-1311, with an overall average lethality of 85% at day 2 postinfection. They thus could be considered virulent isolates. The lethality of strains CIRMBP-1212, CIRMBP-1281, CIRMBP-1283, and CIRMBP-1287 was intermediate and higher than that observed for the least virulent strains CIRMBP-1294 (*P* values of between 0.026 and 0.064) and CIRMBP-1320 (*P* values of between 0.026 and 0.073). Close analysis of the gene contents of the 10 strains exhibiting virulence and the 3 strains considered nonvirulent in the CELA identified 33 genes, 18 of which were already found to be enriched in clade E isolates and 6 of which were enriched in avian clinical isolates. The predicted function of these genes indicates a bias in favor of carbohydrate transport and metabolism (Table S3D).

In line with the data obtained for biofilm robustness, binding to collagen, and growth in chicken serum, the clear gradient of virulence observed in the CELA for clinical isolates supports the hypothesis of the multifactorial nature of *E. cecorum* pathogenesis.

## DISCUSSION

*E. cecorum* has emerged as an opportunistic pathogen in poultry worldwide. The present study shows the clonality of a large collection of clinical *E. cecorum* isolates collected between 2007 and 2017 in the leading French commercial broiler-producing area. It identifies the main phylogenetic clades and subclades and provides the first insight into the intercontinental clonality of clinical isolates of *E. cecorum* from poultry. Six genes significantly associated with the origin of the isolates allow the discrimination of 94% of the avian clinical isolates of the collection from the nonclinical ones. Based on the new complete genomes, we also provide insight into the diversity of the mobile genetic elements of *E. cecorum* that carry ARGs.

One way of assessing the diversity of a species is to analyze its core and accessory genomes (44–46). Although they should be considered rough estimates due to the use of draft genomes, the current *E. cecorum* core genome represents only 14.1% (1,207/8,523 CDSs) of the pangenome and ~50% of the average *E. cecorum* genome. While the size of the core genome is within the range of the one reported previously by Sharma et al., the *E. cecorum* pangenome is now 35% larger than previous estimates due to the addition of 130 genomes to the 18 genomes used previously (28). The increase in the genome size corresponds to an average of only 20 genes per genome; however, the low gene discovery rate is probably due to the high proportion (93%) of clinical poultry isolates in clade E. Indeed, clade E isolates (*n* = 117) accounted for 22% (1,873/8,523) of the accessory genes, while more distantly related isolates of clades A (*n* = 2), B (*n* = 5), C (*n* = 16), and D (*n* = 8) accounted for 34.0% (2,897/8,523) of the accessory genes. The proportions of core genes and accessory genes strongly correlate with the lifestyle of the bacterium. A small core genome compared to a large pangenome reflects the diversity of hosts and lifestyles, as observed for E. coli *sensu stricto* and Salmonella enterica, two ubiquitous species with commensal or pathogenic lifestyles whose core genomes account for 0.39% and 1.9% of the pangenome, respectively (47, 48). Conversely, a high proportion of core genes reflects more restricted lifestyles, such as those of Bacillus anthracis (65%), an obligate pathogen, and Staphylococcus aureus (36%) and Streptococcus pyogenes (37%), two human-restricted pathogens (45). Broader sampling from different hosts and countries is needed to further evaluate the diversity of the *E. cecorum* species.

Genome reduction associated with pathogenicity is observed in many bacteria, including Streptococcus suis and Streptococcus agalactiae, whose genome sizes are reduced in virulent host-adapted isolates (49–52). Although longer *E. cecorum* genomes belonged to clinical poultry isolates, the average genome sizes were similar for all poultry isolates regardless of their clinical status (2.40 ± 0.14 and 2.36 ± 0.11 Mbp for clinical poultry isolates and nonclinical poultry isolates, respectively). This observation differs slightly from those of two previous studies on isolates from the United States, where clinical isolates had shorter genomes than nonclinical isolates (19, 28). The latter is most likely due to the small number of genomes examined and the sampling of clinical isolates, which, according to our findings, all belong to the same phylogenetic subclade, E12. In the current data set, the smallest genomes correspond to isolates from phylogenetic subclade E11, and the largest genomes correspond to isolates from subclade E4. Interestingly, all but one of the E4 genomes (*n* = 27) have no CRISPR-*cas* systems, correlating with abundant ICEs, GIs, and prophages.

Previous molecular studies, using mainly PFGE, have converged toward the genetic homogeneity of *E. cecorum* clinical isolates from the same country compared to nonclinical isolates (19, 22–28). Despite more than 6,500 cases of *E. cecorum* infections in poultry reported in France since 2007 (53), the genetic diversity of *E. cecorum* in France and the genetic relatedness with isolates from other countries have never been studied. Phylogenetic analysis of 100 isolates spanning the period from 2007 to 2017 showed that a single clade (E) was responsible for almost all (96%) cases in farms between 5 and 90 km apart in Brittany. The phylogenetic congruence of clinical isolates from the United States is consistent with two hypotheses: (i) isolates were issued from transcontinental dissemination or (ii) isolates from different regions suffered comparable selective pressures. The use of a limited number of commercial genetic lines of broiler chickens may have contributed to the selection of *E. cecorum* clones with pathogenic potential and allowed transcontinental dissemination. While the hypothesis of the transcontinental spread of clade E isolates through the meat trade is unlikely, trade of live animals (54), surface-contaminated eggs, or transport by wild birds may well have contributed to this spread. The second hypothesis, although less likely, is parallel convergent selection due to breeding conditions. The isolates in this study may not represent the full diversity of the French clinical population of *E. cecorum*; however, their temporal distribution reflects the spread and persistence of a clade particularly adapted to broilers with the emergence over time of some more successful subclades. This is illustrated by the dominance of subclade E4 (82%) in France and subclade E12 (75%) in the United States between 2008 and 2010 in the current data set. Subsequently, the dominant subclades were E6, E13, E11, and E10, the latter two of which were dominant on French farms in 2016. The temporary circulation of specific subclades may be due to natural evolution or adaptive changes in response to modifications of breeding practices (like novel biocides and cleaning procedures or different feed origins, compositions, or additives), but the reasons remain to be determined. Although less frequent, clinical isolates were also found in clades C and D, which contain nonclinical isolates and display higher diversity. Noticeably, isolates of these two clades have multiple ARGs, which may confer a selective advantage and thus may contribute to the pathogenic potential under specific conditions that remain to be determined. Additional genomes of nonclinical isolates but also clinical isolates from diverse countries and other poultry species and husbandry systems are required to obtain a comprehensive view of the *E. cecorum* population structure and determine whether *E. cecorum* clade E isolates are broiler specific.

*E. cecorum* has occasionally been involved in human infections (55–57). Four of the six clinical human isolates in this study were clade E isolates, supporting a poultry origin. However, this does not lead to the conclusion that contamination was foodborne. The two other clinical human isolates belong to clade A and are phylogenetically close. Both have large clusters of highly specific genes, including an iron transporter and an ~60-kbp motility locus characterized by flagellar and chemotaxis genes, which may have been acquired by horizontal gene transfer from other species of enterococci such as Enterococcus casseliflavus, E. gallinarum, or E. columbae encountered in birds but also other animals, including humans, insects, and aquatic hosts for the first two (58).

The dominance of *E. cecorum* clinical isolates from clade E strongly supports the hypothesis that clade E isolates have acquired properties that increase their fitness and/or infectivity. At the core-genome level, all clade E isolates share SNPs that may confer a selective advantage to the host. Of the two nonneutral mutations, one is in the phage shock protein gene *pspC* that encodes an ortholog of the transmembrane protein LiaY, probably involved in resistance to cationic antibiotics and antimicrobial peptides (59). Codon changes leading to synonymous SNPs may also modify the translation efficiency as such to foster cell fitness (60). In addition to the clade E-specific mutations in the core genes, 83 accessory genes were found to be enriched in the clade E isolates, of which 13 genes are of the capsule operon. The capsule is an important virulence factor to evade host immunity, including for enterococci (see reference 61 for a review on the subject). We identified two closely related capsule loci in 81.2% of the clade E isolates. Similarly, Borst et al. (19) identified in the three clinical isolates (SA1 to SA3) of their study a capsular polysaccharide locus, which corresponds to that of the CIRMBP-1228 genome found most frequently in the clade E isolates. The strong association between these capsule loci and the clinical isolates suggests a role in virulence, probably by promoting immune evasion (62). However, virulence, especially for opportunistic pathogens, is a multifactorial process involving multiple bacterial traits such as metabolic functions and stress resistance (63, 64). Other clade E-enriched genes may give *E. cecorum* alternative metabolic capacities to survive and/or multiply in the host. The predicted galactitol phosphotransferase system (CIRMBP1228_02729 to CIRMBP1228_02731), galactonate catabolism enzymes (CIRMBP1228_02728 and CIRMBP1228_02732), as well as the biotin biosynthesis genes may give *E. cecorum* an advantage in competing with commensal species in nutrient-limited environments such as the gastrointestinal tract, considered a portal of entry during the first week of life of the host (10). Of note, the mannitol-1-phosphate 5-dehydrogenase (*mtlD*) gene proposed to be specific for nonclinical isolates (19) was not discriminatory between nonclinical and clinical poultry isolates in our collection because it was present in only seven isolates of different origins. Conversely, we selected 6 genes, which, taken together, allow the discrimination of more than 90% of the clinical isolates. The combined detection of these candidate genes in a larger collection of clinical and nonclinical isolates is necessary in order to evaluate their use for the detection of the early carriage of potentially pathogenic isolates, particularly during the first week of life. In contrast, while ascorbate catabolism genes are dispensable in 70% of the clinical avian isolates, they may confer a competitive advantage to avian nonclinical isolates and human clinical isolates.

MGEs, including prophages, are major contributors to the evolution of the gene repertoire. We identified 75 MGEs corresponding to predicted ICEs or related elements and GIs in the completely sequenced genomes and a total of 105 complete prophages in the 118 sequenced genomes. Three GIs have a composite structure with phage genes and more than one integrase gene, likely resulting from the independent integration of different MGEs (38, 65). *E. cecorum* MGEs are integrated into the 3′ ends or the intergenic regions of genes encoding a ribosomal protein (*rpsB*, *rpsI*, *rpsF*, and *rpmE*) and a few tRNA genes or riboswitches (*tRNA-Thr*, transfer-messenger RNA [*tmRNA*], and *preQ1*) but also various other intergenic regions. This is consistent with the different site specificities of the prevalent integrases of the tyrosine and DDE recombinase families (36, 38, 39), although there is relatively little integration into the tRNA genes that are frequently targeted by tyrosine integrases in streptococci (66). The apparently nonrandom distribution of the *E. cecorum* MGEs integrated relatively far away from the chromosomal origin of replication may be related to the eviction of highly expressed genes located near the origin of replication (67, 68). Among the prophages that we identified, prophages homologous to PHAGE_Strept_5093, PHAGE_Entero_EFC_1, and PHAGE_Bacill_phBC6A52 were also detected in clinical and nonclinical poultry isolates from the United States (28). In contrast, and with the exception of Tn*917*, the ICEs and GIs identified in the French isolates are not well conserved in the U.S. isolates, indicating that they were acquired separately and contributed to local adaptation. In addition to the type IV secretion system involved in the formation of the DNA translocation channel, ICEs encode cell surface adhesins for attachment to the target cell. These include LPxTG cell wall-anchored adhesins such as S. agalactiae antigen I/II family adhesins, also referred to as group B Streptococcus surface proteins (Bsps) (69, 70). These structural proteins were also shown to promote biofilm formation, interactions with host cells, and virulence (71, 72). We identified Bsp-like proteins in Tn*GBS1*- and Tn*GBS2*-related ICEs, proteins containing CnaB domains initially found in microbial surface components recognizing adhesive matrix molecules (MSCRAMMs), and VaFE repeat-containing surface-anchored proteins in diverse *E. cecorum* ICEs and GIs, yet none of these were associated with a specific trait or origin. However, the variability of these adhesins may hinder any association, as they may also compensate for each other. ICEs and GIs may carry diverse genes, known as cargo genes, that are not involved in gene transfer but may confer a selective advantage to the host strain. We have identified several *E. cecorum* cargo genes encoding toxin-antitoxin modules that function as MGE addiction systems but are also involved in the control of bacterial growth (73). Other cargo genes encode restriction-modification systems that protect the cell against horizontal gene transfer or are genes involved in protection against oxidative stress or resistance to cadmium or arsenate, which could confer better fitness contributing to ecological adaptation. An accessory SecA2-SecY2 operon was also identified in strain CIRMBP-1228. Such systems are dedicated to the export of glycosylated serine-rich repeat proteins (SRRPs) that participate in adhesion to host cells and/or biofilm formation (74, 75). Functional analysis of a few selected strains is required to evaluate whether and how these accessory genes contribute to adaptation to environmental challenges.

ARGs are other clinically important cargo genes spread by ICEs and GIs (76). The ARGs identified in *E. cecorum* confer resistance to tetracycline, macrolides, bacitracin, aminoglycosides, and, much more rarely, glycopeptides. The most prevalent ARGs are the *tet*(M), *tet*(L), and *erm*(B) genes. This is in line with the high prevalence of resistance to tetracycline and erythromycin in *E. cecorum* found in various studies, as reviewed by Jung et al. (12), and the use of tetracyclines and macrolides despite substantial efforts to reduce their use in veterinary medicine. As anticipated from the literature, *tet*(M) is carried on ICEs of the Tn*916* family, and *erm*(B) is carried on Tn*917* (77). Other macrolide resistance genes such as *mef*(A), *msr*(D), and *lnu*(B) and aminoglycoside resistance genes such as *ant(6)-Ia* and *aph(3′)-III* are prevalent in U.S. isolates (28). The *mef*(A), *msr*(D), *vat*, and *erm*(C) genes are located on the same GI in CIRMBP-1246 (CIRMBP1246_01012 to CIRMBP1246_01050), *lnu*(C) is adjacent to the IS*1595* family transposase IS*Sag10*, and the two adjacent genes *lnu*(B) and *lsa*(E) are next to the IS*1595* family transposase IS*Cpe8*, previously described in an avian Clostridium perfringens strain carrying the lincomycin resistance gene *lnu*(P) on the plasmidic transposable element tIS*Cpe8* (78). Another prevalent ARG is the bacitracin resistance operon *bcr* in isolates of clades C and D and subclades E10, E11, and E12. This operon is frequently associated with the *tet*(M) and *tet*(L) genes on ICEs of the Tn*916* family. The highly conserved nucleotide sequence of the *bcr* operon, including the flanking element IS*Enfa1*, and its location on Tn*916*-like elements or GIs are consistent with avian interspecies transmission involving E. faecalis, E. faecium, and C. perfringens (79, 80). Note that the carriage of *tet*(M) and *tet*(L) on the same Tn*916*-like element is uncommon. It was first described in Streptococcus gallolyticus and was proposed to benefit the host bacterium under stressful conditions (81, 82). A single clinical poultry isolate from France has the *vanA* operon, a gene previously described in an *E. cecorum* strain from retail poultry in Japan (83). Overall, 26% (*n* = 39) of the isolates carry multiple ARGs (4 to 10) conferring resistance to at least three antimicrobial families and are prevalent in clinical and nonclinical isolates of clades C and D and subclades E11 and E12. The very few strains without predicted ARGs and the differential ARG profiles between French and U.S. isolates probably reflect strong antibiotic selective pressure that differs between the two countries. Indeed, in-feed bacitracin and in-feed macrolides are still used in poultry farming in the United States (84). The successive European bans of antibiotics (avoparcin in 1997; bacitracin, spiramycin, tylosin, and virginiamycin in 1999; and avilamycin and flavophospholipol in 2006) and French national EcoAntibio plans (85, 86) launched in 2012 and 2017 to fight antimicrobial resistance in animal health and promote the responsible use of antibiotics might have contributed to containing the spread of ARGs and reducing MLS resistance genes, as observed in recent isolates of subclade E10. In fact, this is in line with the decreasing trend of macrolide resistance of *E. cecorum* strains isolated from French poultry according to the French Surveillance Network for Antimicrobial Resistance in Bacteria from Diseased Animals (RESAPATH) (https://shiny-public.anses.fr/resapath2/). However, there has been a marked increase in bacitracin resistance genes in French isolates since 2015, even though bacitracin is not used in avian veterinary medicine in France (87). Bacitracin is produced by Bacillus licheniformis and Bacillus subtilis strains. With the need for alternatives to antibiotics in livestock, bacillus strains are used as probiotics or applied together with lactic acid bacteria as a protective biofilm against pathogens (88–90). The increase in *E. cecorum* isolates carrying the *bcr* operon points to the need to examine whether *Bacillus* strains applied on farms produce bacitracin or related antimicrobial compounds that could contribute to the dissemination of the *bcr* operon. Reassuringly, relatively few aminoglycoside and vancomycin resistance genes that target gentamicin and glycopeptides, two critically important antimicrobials in human medicine, are found in French isolates, as elsewhere in Europe (12).

Overall, the results of this study shed light on the population of *E. cecorum* clinical isolates in France and reveal a genetic linkage with *E. cecorum* clinical isolates from elsewhere. We have shown that based on the available data, the majority of clinical poultry isolates are phylogenetically distinct from nonclinical poultry isolates and form a main clade responsible for the outbreaks of *E. cecorum* in France and probably the United States and Europe. ICEs and GIs are the main carriers of antimicrobial resistance. The E clade of *E. cecorum* appears to have adapted to the conditions under which poultry are reared, highlighting its importance as an emerging threat to the poultry industry worldwide. This information can be used to design and guide preventive strategies to reduce the impact of *E. cecorum* clade E isolates.

## MATERIALS AND METHODS

### Bacterial strains.

A total of 118 strains were collected from various laboratories and deposited at the International Center for Microbial Resources—Bacterial Pathogens (CIRM-BP) (https://www6.inrae.fr/cirm_eng/BRC-collection-and-catalogue/CIRM-BP) (see Table S1 in the supplemental material). The majority of them (*n* = 100) were isolated between 2007 and 2017 from diseased birds from 16 broiler farms located in Brittany, France, and were provided by Labofarm (Loudéac, France). Other poultry strains were isolated in Poland (*n* = 5), Belgium (*n* = 1), and the United States (*n* = 6). Six strains from human infections were isolated in France (*n* = 3), Belgium (*n* = 2), and Germany (*n* = 1). The sources and the original names of the strains from abroad are indicated in Table S1. Additional details are available in the supplemental material.

### Genome sequencing and analysis.

All genomic DNA was subjected to random shotgun library preparation using the TruSeq DNA PCR-free kit (Illumina). Ready-to-load libraries were sequenced on an Illumina MiSeq or HiSeq 3000 platform at GeT-PlaGe (Toulouse, France) and a HiSeq 2500 platform at Eurofins Genomics (Germany) using 150-bp paired-end chemistry. DNA of 14 isolates was also sequenced using Oxford Nanopore Technology platforms. The preparation of libraries and sequencing were performed at the GeT-PlaGe core facility (INRAE, Toulouse, France) or MetaGenoPolis (INRAE, Jouy-en-Josas, France) according to the manufacturer’s instructions. Additional details are available in the supplemental material.

The 104 genomes with only Illumina reads were assembled using RiboSeed v0.4.73 (91). RiboSeed uses a reference genome to resolve rRNA operons and globally improve the whole-genome assembly. Assemblies were performed using NCTC12421 (GenBank accession number NZ_LS483306) as the reference genome for rRNA operons, using SPAdes v3.13.0 (92) as the assembler in “careful” mode with *k* values of 21, 33, 55, 77, and 99. The 14 genomes with Illumina and Nanopore reads were assembled using Unicycler 0.4.4 (93), an assembly pipeline for bacterial genomes that uses SPAdes for short-read assembly and Miniasm and Racon for long-read assemblies and polishing. Unicycler was launched with default parameters. Genomes were compared using Mummer 3.2.3 (94) with parameter to compute all maximal matches (-maxmatch) and a minimal length of match of 100 nucleotides (-l parameter). Inversions in the genomes were checked by realigning long reads on the assembly using minimap 2.2.17 (95). The veracity of the assembly on the breaking points of the inversion was manually checked by visual inspection of the alignment of the long reads encompassing the inversion.

Genome annotation was conducted with Prokka v1.12 (96). First, coding DNA sequences were identified on contigs longer than 200 bp by Prodigal v2.6.1 (97), which penalized CDSs shorter than 250 bp in order to filter out false positives. CDSs were first annotated (–proteins Prokka parameter) using a protein bank extracted from all of the complete *Enterococcus* genomes in the RefSeq database (4,198 genomes retrieved in April 2020). Annotations from hits with an E value cutoff of 10^−9^ and 80% coverage were transferred. CDSs with no hits on this bank were annotated using the Prokka default workflow and data banks.

### Pangenome analysis and phylogenomic tree construction.

Pangenome analysis was performed by comparing 118 *de novo*-sequenced genomes of *E. cecorum* and 30 public genomes from the NCBI with *N*_50_ values above 20 kb (January 2021). One reference genome was available in the NCBI nucleotide database, NCTC12421. Protein clustering was performed by Roary (98) v-3.12.0 with 94% identity, a “percentage of isolates a gene must be in to be core” of 100%, and the parameter “without split paralogs.” Genes classified as core were genes present in all 148 genomes. Accessory genes were all other genes present in 147 or fewer genomes. The gene accumulation curves were produced with ggplot2 (99) from Roary data. The phylogenomic analysis was performed using the *E. cecorum* core genome. The 1,207 core genes of *E. cecorum* were aligned using a codon-aware alignment produced by PRANK (v170427); an unrooted tree was then constructed using the BioNJ algorithm (100) in SeaView (v4.2) (101), using the Jukes-Cantor distance and 1,000 bootstrap replicates. Clades were determined using the Jukes-Cantor distance between aligned core genes of <0.021 and a bootstrap value of >75%. The graphical representation of the phylogenetic tree was performed with iTOL (v4) (102).

### Acquired antimicrobial resistance gene search.

ARG research was performed using the ResFinder (v2.1) (103) tool and database (26 April 2019) for 90% gene identity and 60% coverage. BLASTN was used to detect the *bcr*-like gene (*uppP_2*) and to compare the *vanA* locus with pVEF3 of E. faecium 01_233 (34). Positive hits had at least 90% nucleotide identity and 60% coverage. GenoPlotR (v0.8.11) was used to visualize BLASTN results with >90% identity (34).

### ICE, GI, and prophage detection.

ICEs and IMEs were detected in the 11 complete genomes and the 2 large contigs of the genome of CIRMBP-1320 using ICEscreen (https://icescreen.migale.inrae.fr) and then inspected visually for delineation. Genomic islands corresponded to large insertions of more than 10 CDSs resulting in a synteny break between two genomes. Prophage prediction was performed using the prophage detection tools PHASTER (Phage Search Tool, Enhanced Release) (104) and VIBRANT (Virus Identification by Iterative Annotation) (105). Only predicted prophages with a prediction score of ≥100 with PHASTER or VIBRANT were retained and manually inspected to determine the attachment and integration sites in reference to the NCTC12421 genome (GenBank accession number NZ_LS483306). PHASTER was further used to identify the most similar phage genomes.

### Hierarchical clustering.

Estimated marginal means of biovolumes of biofilm and adhesion to collagen and serum growth indices were adjusted for experiment and strain factors using a linear model with the emmeans R package (1.4). Strains with similar Euclidian distances between estimated marginal means were grouped using a hierarchical clustering algorithm with average linkages. Clusters of strains were defined by cutting the dendrogram dynamically using the DynamicTreeCut R package (1.63-1) (106).

### Genome-wide association study.

Single nucleotide polymorphism analysis was performed using GATK version 4.4.2 (107) with core genes of NCTC12421 as the reference. Default parameters were applied with the HaplotypeCaller module to call SNPs. GWASs were performed using TreeWas (1.0) (108) to identify genetic loci (SNPs obtained from GATK analysis and gene presence/absence obtained from Roary analysis) associated with clade membership (clade A, B, C, D, or E), clinical origin (clinical and nonclinical poultry isolates and clinical human isolates), biofilm robustness (binary strong values versus others, binary weak values versus others, and phenotypic groups obtained by clustering), and collagen type II adhesion and/or growth in chicken serum (similarly to biofilm robustness). Significant genetic loci corresponded to a *P* value of ≤0.05 according to a terminal test.

A set of criteria was applied to the TreeWas output to select clade-specific SNPs or enriched genes. The criteria applied to select SNPs were more stringent, and only clade-specific SNPs were retained (sensitivity equal to 1 or 0 and specificity equal to 1 or 0). Genes whose presence was associated with clade membership (clade A, C, or E) or clinical origin (clinical and nonclinical poultry isolates and clinical human isolates), biofilm robustness, adhesion to collagen type II, or growth in chicken serum had sensitivity and specificity scores above 0.66. In addition, genes whose absence was associated with clade membership or clinical origin, biofilm robustness, adhesion to collagen type II, or growth in chicken serum had sensitivity and specificity scores below 0.33.

### Data availability.

All genomic data have been deposited in the EMBL ENA database under project accession number PRJEB50514. Accession numbers of raw reads and assembled genomes are available in Table S2A.
