# Supplementary figures and images for: Comparative Genome Analysis of Enterococcus cecorum Reveals Intercontinental Spread of a Lineage of Clinical Poultry Isolates
Source: mSphere. 2023 Feb 16;8(2):e00495-22. doi: 10.1128/msphere.00495-22 (PMC10117131; doi:10.1128/msphere.00495-22)

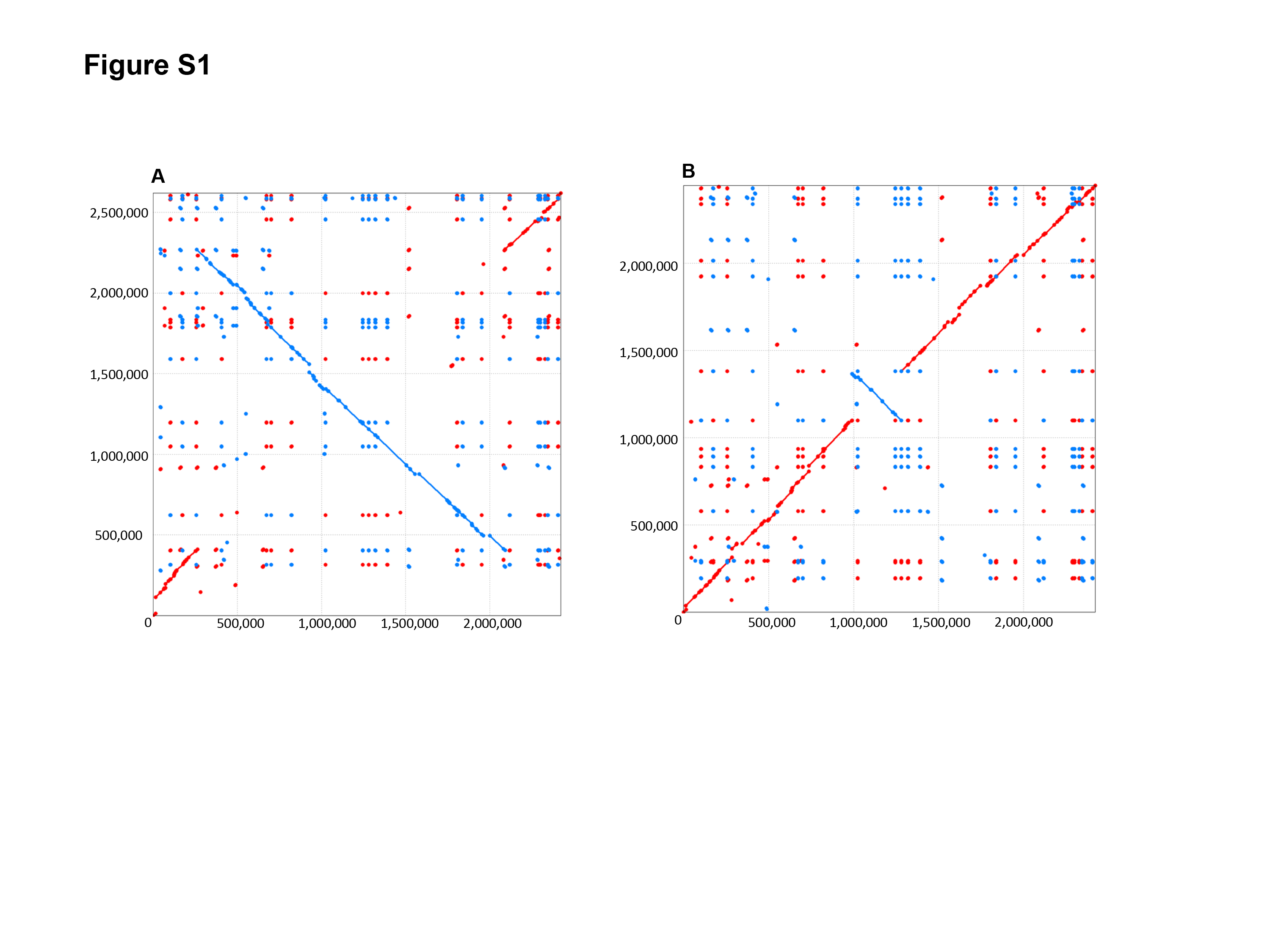

Supplement: FIG S1 [file msphere.00495-22-s0001.tif]

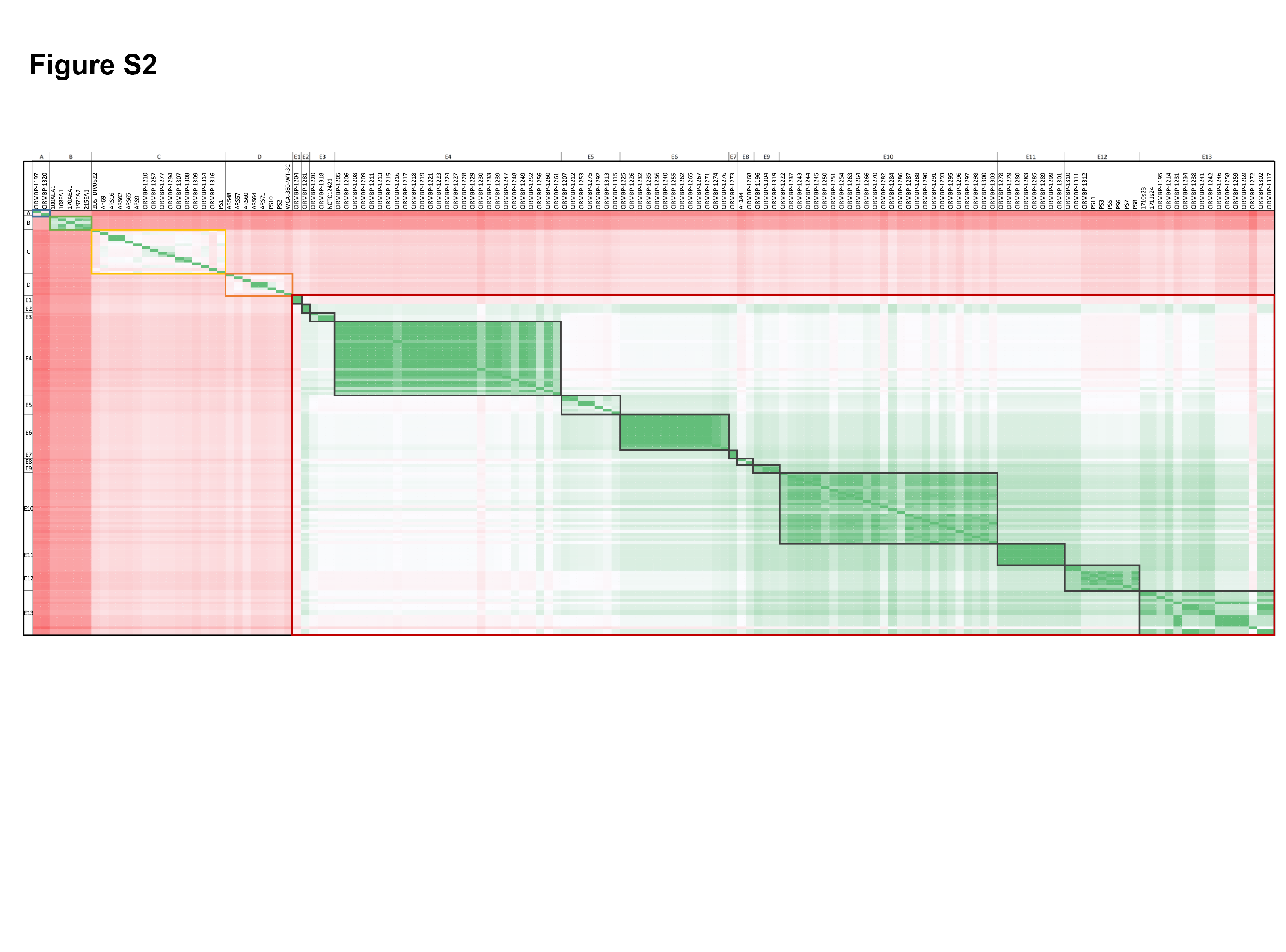

Supplement: FIG S2 [file msphere.00495-22-s0002.tif]

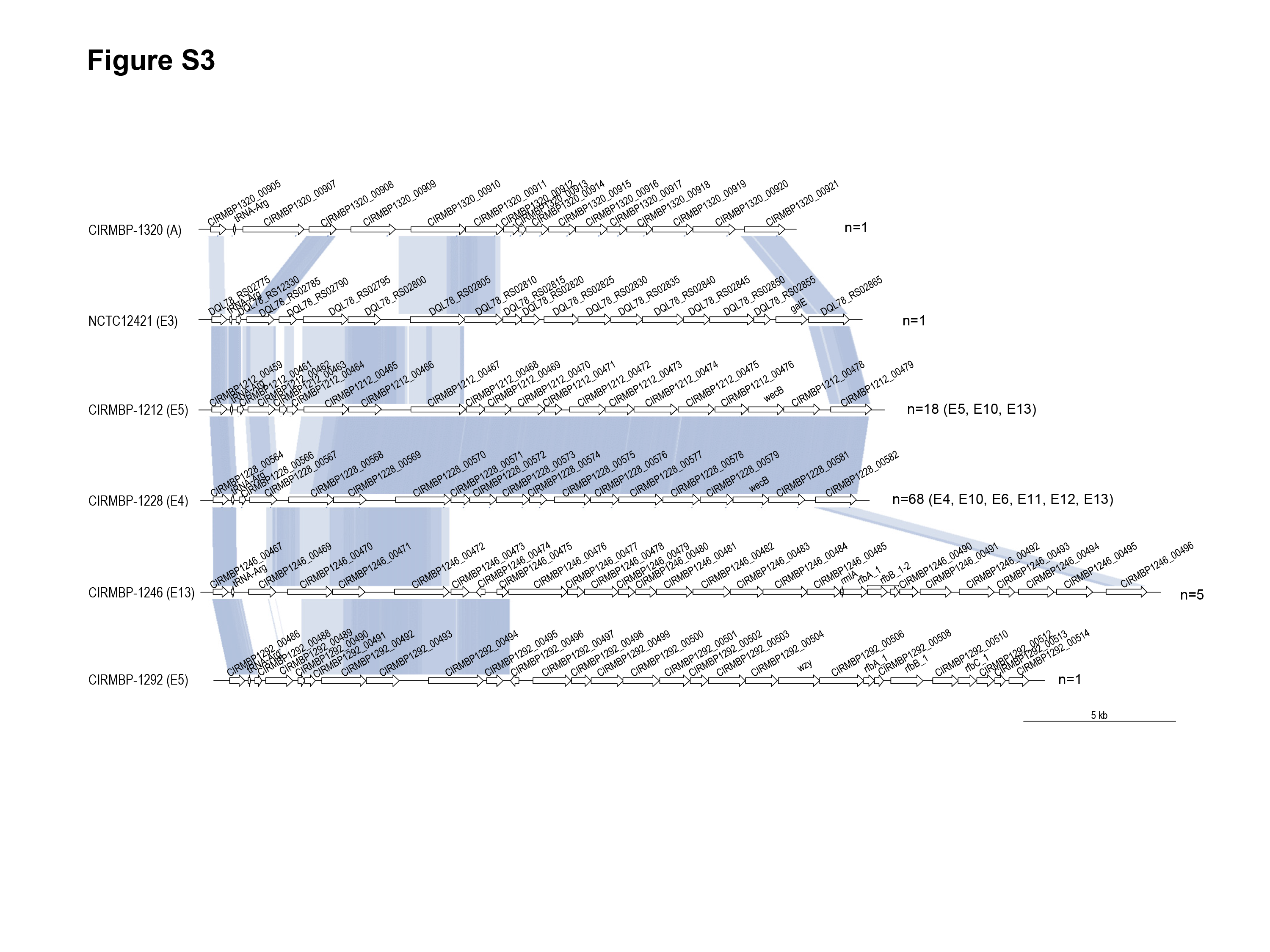

Supplement: FIG S3 [file msphere.00495-22-s0003.tif]

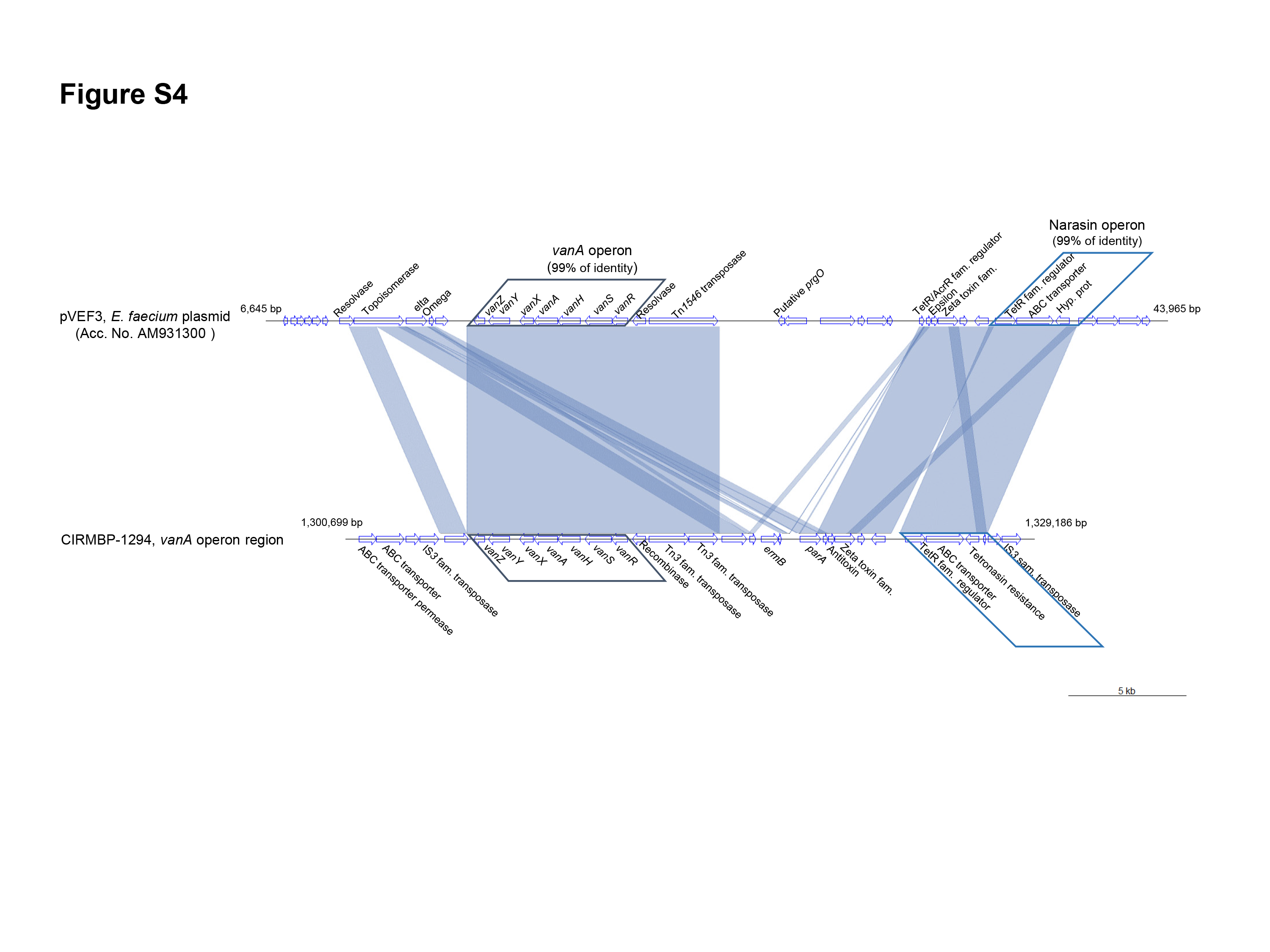

Supplement: FIG S4 [file msphere.00495-22-s0004.tif]
