# Supplementary material for: Comparative Genome Analysis of Enterococcus cecorum Reveals Intercontinental Spread of a Lineage of Clinical Poultry Isolates
Source: mSphere. 2023 Feb 16;8(2):e00495-22. doi: 10.1128/msphere.00495-22 (PMC10117131; doi:10.1128/msphere.00495-22)
Supplement: TEXT S1 [file msphere.00495-22-s0010.pdf]

Supplemental material

Methods

Strains, storage and availability

The eighteen strains that were isolated from poultry were obtained from the SGGW-Faculty of Veterinary Medicine (University of Life Sciences, Warsaw, Poland; n=5) and from the North Carolina State College of Veterinary Medicine (Raleigh, USA, n=6). Six strains isolated from human infections were obtained from IRSD (University of Toulouse, INSERM, INRAE, ENVT, UPS, Toulouse, France; n=3), Ghent University Hospital (Ghent, Belgium; n=1), the AZ Sint-Lucas Hospital (Ghent, Belgium; n=1), and the Institute of Medical Microbiology, Virology, and Hygiene (Rostock University Medical Center, Rostock, Germany; n=1). The type strain was obtained from the DSMZ-German Collection of Microorganisms and Cell Cultures (Leibniz, Germany). All the isolates had been isolated prior to this study. The clinical poultry isolates were obtained from routine diagnostic performed by veterinary laboratories. The nonclinical poultry isolates were obtained from laboratories applying procedures approved by institutional animal ethics committees. The human isolates were collected from patients who gave informed consent in accordance with institutional ethical procedures.

Strains were stored frozen in liquid nitrogen and -80°C in BHI broth with 15% glycerol and freeze-dried in Greaves solution (1) and maintained at 4°C. The purity of each strain was checked by microscopic and macroscopic observations and the identity of each strain was validated after storage by API Rapid 32 Strep gallery and MALDI TOF Mass Spectrometry (Bruker). To ensure the proper implementation of the Nagoya Protocol when using strains from abroad, the national focal point of each country was contacted through the Access and Benefit-Sharing Clearing-House (ABSCH) platform (https://absch.cbd.int/). When the original laboratory agreed, the strains and their associated data were deposited at the International Center for Microbial Resources dedicated to Bacterial Pathogens (CIRM-BP, https://www6.inrae.fr/cirm_eng/BRC-collection-and-catalogue/CIRM-BP) to be made available to the scientific community for research purposes.

DNA preparation

Strains from frozen stocks were grown overnight in 5 ml Brain Heart Infusion broth (BHI, DIFCO) supplemented with 5% horse serum (Gibco) and incubated at 37 °C under 5% CO_2. Overnight cultures were diluted 20-fold in 10 ml of BHI-serum and incubated under the same conditions until the culture reached an optical density of 1.2 at 600 nm (OD_{600}). Bacteria from 5 ml of the culture were centrifuged (Eppendorf Centrifuge 5804R) for 6 minutes at 5,000 g. The bacterial pellet was then washed in 5 ml of Tris 10 mM, pH 7.5 and supernatant was discarded after 6 minutes of centrifugation at 5,000 g. Total bacterial DNA was extracted and purified using the Nucleobond AXG kit (Macherey Nagel, Hoerdt, France) AXG20 column and NucleoBond Buffer Set III. Cell lysis was performed by resuspending the bacterial pellet in G3 buffer containing 200 µg/ml RNase A, 10 mg/ml lysozyme (Sigma and 70 U/ml mutanolysin (Sigma), and incubated for 45 - 60 minutes at 37 °C. The lysis was prolonged by adding 0.44 mg/ml proteinase K and incubating at 56°C for 10-15 minutes. DNA was then extracted as according to the manufacturer's instructions. DNA was quantified using a Quantifluor ONE dsDNA system and a Quantus fluorometer from Promega according to the instructions of the manufacturer. DNA quality control was assessed on a 2100 Bioanalyzer system (Agilent Technologies).

Sequencing, quality control and genome assembly

At each step, DNA was quantified using the Qubit dsDNA HS Assay Kit (Life Technologies, ville). DNA purity was tested using the Nanodrop (Thermo Fisher Scientific), and size distribution and degradation assessed using the Fragment analyzer (AATI) and the High Sensitivity DNA Fragment Analysis Kit. Purification steps were performed using AMPure XP beads (Beckman Coulter). For 1 Flowcell, 4 µg of each DNA (13 samples) was purified then 2µg were sheared at 23 kb/Speed 31 using the Megaruptor 3 system (Diagenode). The DNA of CIRMBP-1246 (1.5 µg) was sheared at 8-9kb using G-Tube (Covaris) twice for 30 seconds at 7,200 rpm each. A one step DNA damage repair + END-repair + dA tail was performed on 1 µg of each sample. Then specific indexes were ligated to each sample. The library was generated by an equimolar pooling of these barcoded samples. Then adapters were ligated to the library

which was loaded on a R9.4.1 flowcell and sequenced on a GridION instrument (Oxford Nanopore Technologies Ltd.) at 20 fmol within 72 h. The library of CIRMBP-1246 DNA was loaded onto a FLO-MIN106 R9.4.1 flow cell and run on a MinION Mk1B instrument (Oxford Nanopore Technologies Ltd.) for 48 h.

Illumina read quality was controlled with FastQC v0.11.5 (2). Reads were sampled randomly to have only between 100 and 200X of sequencing depth for the assembly. Quality of Nanopore reads was checked with NanoPlot 1.29.1 (3). The number of bases per sample ranged from 1.6 Gb to 2.4 Gb with a median read length of 13kb. The 104 genomes with only Illumina reads were assembled using RiboSeed v0.4.73 (4). RiboSeed uses a reference genome to resolve ribosomal RNA operons and globally improve whole genome assembly. Assemblies were performed using NCTC 12421 (# NZ_LS483306) as reference genome for rRNA operons, using SPAdes v3.13.0 (5) as assembler in "careful" mode using k values of 21, 33, 55, 77 and 99.

Biofilm formation

Biofilm formation was quantified as previously described by Bridier al. (6). Two hundred µl of an overnight culture in Todd Hewitt broth (BD) with 1.0% yeast extract (THY), adjusted to ~2x10^6 CFU/ml, were added in each well. The microtiter plate was then incubated at 37 °C under 5% CO_2 for 90 min to allow adhesion of the bacteria. Wells were rinsed and refilled with THY. The plates were incubated at 37°C under 5% CO_2 for 24 h. To consider only the cohesive fraction of the biofilm, weakly bound cells were removed by rinsing the wells with NaCl 0.9% with an automatic plate washer (Wellwash Versa Microplate Washer, Thermo Fisher Scientific). Residual biofilms were stained using the cell permeant nucleic acids dye SYTO 9 (Life Technologies) diluted at 5 µM in NaCl 0.5%. Images were acquired using a Leica SP8 confocal laser scanning microscope (Leica Microsystems). The green SYTO 9 emitted fluorescence was recorded on hybrid detectors between 500 and 550 nm after excitation at 488 nm with an Argon laser. For each well, 4 randomly chosen fields were scanned and 3D images were reconstructed from Z-stacks (55 images of 512 x 512 pixels each z-step) using Imaris v9.3 software (Bitplane). Biovolume (µm^3/µm^2), representing the

overall volume of cells (μm^3) in the observation field (here 14,209 μm^2), was calculated directly from the raw images using Image J software and Comstat 2 plugin (7). Each strain was tested in duplicate or triplicate.

Adhesion to type II collagen

Polystyrene 96-well plates (Greiner Bio One, µClear) coated with chicken type II collagen (C9301, Sigma, France) were prepared as described previously (8); 50 µl of a 40 µg/ml type II collagen solution were incubated overnight at 4 °C. After 3 washes with PBS, wells were saturated with 200 µl of 1% (w/v) bovine serum albumin (Sigma) at room temperature for 90 min, followed by 3 washes with PBS-BSA 0.1%. Overnight cultures of *E. cecorum* were pelleted at 3,000 g for 8 min and washed twice in PBS before dilution to ~$2x10^7$ CFU/ml. 100 µl of bacterial suspension were added in each well of a collagen-coated polystyrene plate. After 90 min of adhesion at 37°C under 5% CO_2, wells were rinsed twice with PBS and once with NaCl 0.5%. The adherent bacteria were stained and quantified in biovolume ($\mu m^3/\mu m^2$) as described above. Each strain was tested in triplicate or duplicate. Wells without bacteria or collagen served as negative controls.

Growth in chicken serum

Growth in chicken serum was determined according to Braga et al, 2016 (9). Chicken serum was prepared from 5 ml of blood white Leghorn SPF chickens, strain PA12. The blood was allowed to clot for a minimum of 4 hours at room temperature prior to centrifugation for 20 min at 1500 g. 50 µl of serum were transferred into 9 ml of BHI broth and incubated at 37°C without shaking for 18 h to check for sterility. Half of the sterile serum was heated at 56°C for 30 min. Overnight *E. cecorum* cultures in THY at 41°C under CO_2 5% were washed in sterile Dulbecco's Phosphate-Buffered Saline (DPBS, Gibco) and diluted 1:100 in DPBS. Each well of a microtiter plate was inoculated with 100 µl of the bacterial suspension and 100 µl of serum or 100 µl of heated serum. The plate was incubated at 41°C under CO_2 5% for 6 h. At t0 and 6h after incubation, 20 µl of each well were sampled to enumerate the bacteria using Easy Spiral on BHI plates, which were read using a Scan 4000 (Interscience). Bacteria used as control were *E. coli* strain BEN2908

(resistant to bactericidal effect of serum) and strain BEN-5052 (sensitive to serum). *E. coli* strains were cultured in LB-Miller broth (MP Biomedicals) at 37°C with shaking. Experiments were performed at least twice. The serum growth index was calculated as follows: (CFU/ml in heated serum / CFU/ml in serum)$_{t6h}$ / (CFU/ml in heated serum / CFU/ml in serum)$_{t0}$. The more the index is > 1, the less the strain is able to grow in the presence of serum. If the index is <1, the strain grows better in the unheated serum, potentially by using a heat-labile component.

Chicken embryo lethality assay (CELA)

E. cecorum free SPF layer type chicken eggs (chicken line PA12, PFIE, INRAE Val de Loire) were incubated at 37.8 °C and 45% humidity with the egg air space up for 12 days. Prior to inoculation, the unfertilized eggs and eggs with dead embryos were removed by candling (10). 1 ml of an overnight *E. cecorum* culture in THY at 41°C under CO_2 5% was washed and resuspended in 1 ml DPBS. Bacteria were plated on BHI using Easy Spiral (Interscience) and counted using a colony counter (Scan 4000, Interscience). Ten eggs for each *E. cecorum* isolate were inoculated with 0.1 ml *E. cecorum* suspension into the allantoic cavity as described previously (10). Ten control eggs were inoculated with 0.1 ml sterile DPBS. All eggs were incubated for 7 days post inoculation and embryonic mortalities were recorded daily for each group by candling.

References

1. Greaves RI. 1960. Preservation of living cells by freeze-drying. Ann N Y Acad Sci 85:723-8.
2. Andrews S. 2010. FastQC: a quality control tool for high throughput sequence data. https://wwwbioinformaticsbabrahamacuk/projects/fastqc/.
3. De Coster W, D'Hert S, Schultz DT, Cruts M, Van Broeckhoven C. 2018. NanoPack: visualizing and processing long-read sequencing data. Bioinformatics 34:2666-2669.

4. Waters NR, Abram F, Brennan F, Holmes A, Pritchard L. 2018. riboSeed: leveraging prokaryotic genomic architecture to assemble across ribosomal regions. Nucleic Acids Res 46:e68.
5. Bankevich A, Nurk S, Antipov D, Gurevich AA, Dvorkin M, Kulikov AS, Lesin VM, Nikolenko SI, Pham S, Prjibelski AD, Pyshkin AV, Sirotkin AV, Vyahhi N, Tesler G, Alekseyev MA, Pevzner PA. 2012. SPAdes: a new genome assembly algorithm and its applications to single-cell sequencing. J Comput Biol 19:455-77.
6. Bridier A, Dubois-Brissonnet F, Boubetra A, Thomas V, Briandet R. 2010. The biofilm architecture of sixty opportunistic pathogens deciphered using a high throughput CLSM method. J Microbiol Methods 82:64-70.
7. Heydorn A, Nielsen AT, Hentzer M, Sternberg C, Givskov M, Ersbøll BK, Molin S. 2000. Quantification of biofilm structures by the novel computer program COMSTAT. Microbiology (Reading) 146 (Pt 10):2395-2407.
8. Chagnot C, Agus A, Renier S, Peyrin F, Talon R, Astruc T, Desvaux M. 2013. *In vitro* colonization of the muscle extracellular matrix components by *Escherichia coli* O157:H7: the influence of growth medium, temperature and pH on initial adhesion and induction of biofilm formation by collagens I and III. PLoS One 8:e59386.
9. Braga JF, Chanteloup NK, Trotereau A, Baucheron S, Guabiraba R, Ecco R, Schouler C. 2016. Diversity of *Escherichia coli* strains involved in vertebral osteomyelitis and arthritis in broilers in Brazil. BMC Vet Res 12:140.
10. Trotereau A, Schouler C. 2019. Use of a chicken embryo lethality assay to assess the efficacy of phage therapy. Methods Mol Biol 1898:199-205.
